# The crosstalk between endothelial cells and vascular smooth muscle cells aggravates high phosphorus-induced arterial calcification

**DOI:** 10.1038/s41419-022-05064-5

**Published:** 2022-07-26

**Authors:** Xiao Lin, Su-Kang Shan, Feng Xu, Jia-Yu Zhong, Feng Wu, Jia-Yue Duan, Bei Guo, Fu-Xing-Zi Li, Yi Wang, Ming-Hui Zheng, Qiu-Shuang Xu, Li-Min Lei, Wen-Lu Ou-Yang, Yun-Yun Wu, Ke-Xin Tang, Muhammad Hasnain Ehsan Ullah, Xiao-Bo Liao, Ling-Qing Yuan

**Affiliations:** 1 National Clinical Research Center for Metabolic Diseases, Department of Metabolism and Endocrinology, The Second Xiangya Hospital, Central South University, Changsha, 410000 China; 2 Department of Radiology, the Second Xiangya Hospital, Central South University, Changsha, China; 3 Department of PET Center, the Xiangya Hospital, Central South University, Changsha, China; 4 Department of Pathology, the Second Xiangya Hospital, Central South University, Changsha, China; 5 Department of Cardiovascular Surgery, the Second Xiangya Hospital, Central South University, Changsha, China

**Keywords:** Cell biology, Calcification

## Abstract

Arterial calcification is highly prevalent, particularly in patients with end-stage renal disease (ESRD). The osteogenic differentiation of vascular smooth muscle cells (VSMCs) is the critical process for the development of arterial calcification. However, the detailed mechanism of VSMCs calcification remains to be elucidated. Here, we investigated the role of exosomes (Exos) derived from endothelial cells (ECs) in arterial calcification and its potential mechanisms in ESRD. Accelerated VSMCs calcification was observed when VSMCs were exposed to ECs culture media stimulated by uremic serum or high concentration of inorganic phosphate (3.5 mM Pi). and the pro-calcification effect of the ECs culture media was attenuated by exosome depletion. Exosomes derived from high concentrations of inorganic phosphate-induced ECs (ECs^HPi^-Exos) could be uptaken by VSMCs and promoted VSMCs calcification. Microarray analysis showed that miR-670-3p was dramatically increased in ECs^HPi^-Exos compared with exosomes derived from normal concentrations of inorganic phosphate (0.9 mM Pi) induced ECs (ECs^NPi^-Exos). Mechanistically, insulin-like growth factor 1 (IGF-1) was identified as the downstream target of miR-670-3p in regulating VSMCs calcification. Notably, ECs-specific knock-in of miR-670-3p of the 5/6 nephrectomy with a high-phosphate diet (miR-670-3p^EC-KI^ + NTP) mice that upregulated the level of miR-670-3p in artery tissues and significantly increased artery calcification. Finally, we validated that the level of circulation of plasma exosomal miR-670-3p was much higher in patients with ESRD compared with healthy controls. Elevated levels of plasma exosomal miR-670-3p were associated with a decline in IGF-1 and more severe artery calcification in patients with ESRD. Collectively, these findings suggested that ECs-derived exosomal miR-670-3p could promote arterial calcification by targeting IGF-1, which may serve as a potential therapeutic target for arterial calcification in ESRD patients.

## Introduction

Arterial calcification is mainly characterized as the accumulation of calcium phosphate salts in the vessel wall, especially medial aortic calcification (also known as Mönckeberg’s calcification). It is a common complication in patients with end-stage renal disease (ESRD), increasing the morbidity and mortality of cardiovascular events [1]. The trans-differentiation of vascular smooth muscle cells (VSMCs) into osteoblast-like cells seems to be a critical pathological process in arterial calcification [2]. However, the mechanism is multifactorial and incompletely understood.

The pathological mechanism involved in arterial calcification in ESRD populations includes Ca/Pi dysregulation, decreased calcific inhibitors, calciprotein particles, abnormalities of microRNA (miRNA), elastin degradation, and so on [3]. Among these, hyperphosphatemia is a critical manifestation of metabolic disorders in ESRD patients. Clinical evidence indicated that coronary artery calcification is independently correlated with hyperphosphatemia in patients with ESRD. The elevated serum phosphate levels, even those in the high-normal range, suggest a higher risk of cardiovascular mortality in chronic kidney disease (CKD) patients [3]. Therefore, we focused on investigating the mechanism of hyperphosphatemia on arterial calcification.

Endothelial cells (ECs) are adjacent to VSMCs in blood vessels, and the interaction between ECs and VSMCs is very important for the physiological and pathological changes to the vasculature [4]. The intercellular communication between ECs and VSMCs often happens via direct contact or paracrine signaling. Recently, most studies have been focused on the function of ECs, as ECs are directly stimulated by the blood compositions, such as hyperphosphataemia, hypertension, hyperlipidemia, and hyperglycemia [5–8]. Nevertheless, arterial calcification mainly occurs in VSMCs of the arterial media layer in patients with ESRD [3, 9]. However, how ECs affect the functions of VSMCs remains unknown.

Exosomes (Exos) are small extracellular vesicles with a diameter of 40–150 nm that originate from endosomal multivesicular bodies and could be released by multiple types of cells, tissues, or organs [10, 11]. Recently, numerous studies have demonstrated that exosomes are critical paracrine mediators of intercellular communication [12, 13] and regulate the functions of recipient cells or tissues by transferring functional molecules, including proteins, lipids, and nucleic acids (mRNA, miRNAs, lncRNAs et al.) [14–16]. Hergenreider et al. reported that ECs could secrete exosomal miR-143/145 clusters to transfer to VSMCs and modulate the formation of atherosclerotic lesions by targeting Krüppel-like factor 2 (KLF2) [15]. Deng et al. also demonstrated that miR-143-3p modulated exosome-mediated pulmonary arterial ECs and VSMCs communication in the pathogenesis of pulmonary arterial hypertension [16]. However, it is unclear whether and how exosomes also mediate communication between ECs and VSMCs in arterial calcification in ESRD.

Here, we investigated the crosstalk between ECs and VSMCs and the role of exosomes derived from ECs in regulating arterial calcification. Firstly, we found that the substance released to ECs culture medium regulated VSMCs calcification under the uremic/high phosphorus environment, and the effect of pro-calcification was considerably blunted after the extracellular vesicles secreted by ECs were removed. Moreover, the high concentration of inorganic phosphate-induced ECs-derived exosomes (ECs^HPi^-Exos) could be taken up by VSMCs both in vitro and in vivo and promoted arterial calcification. Mechanistically, we found the amount of miR-670-3p was dramatically increased in ECs^HPi^-Exos and insulin-like growth factor 1 (IGF-1) was identified as the downstream functional target of exosomal miR-670-3p in the regulation of arterial calcification. Moreover, exosomes also modulated arterial calcification in the 5/6 nephrectomy with a high-phosphate diet (5/6 NTP) mice. Most importantly, ECs-specific knock-in or knock-out of miR-670-3p mice with the 5/6 NTP accompanied by a high-phosphate diet significantly increased or decreased artery calcification. Besides, elevated levels of plasma exosomal miR-670-3p were associated with a decline in plasma IGF-1 in patients with ESRD patients compared with healthy controls. Taken together, our present study uncovered a crucial role of ECs-derived exosomal miR-670-3p in modulating arterial calcification and suggested that miR-670-3p released by ECs may serve as a potential target for therapy and prognosis of artery calcification in patients with ESRD.

## Materials and methods

### Cell culture and transfection

Mice ECs were purchased from CHI Scientific, Inc. (1-3029) with STR certification. Mice VSMCs were isolated from 6 to 8-week-old male C57/BL mice as described before [17]. ECs were cultured in F-12k medium (Hyclone, Logan, USA) supplemented with 12% FBS (Gibco, Invitrogen, New York, USA), penicillin (100 U/mL) and streptomycin (100 μg/mL), 0.05 mg/mL endothelial cell growth supplement (ECGS) and 0.1 mg/mL heparin (Sciencell, San Diego, USA). VSMCs were cultured in Dulbecco’s Modified Eagle’s Medium (DMEM, Hyclone, Logan, USA) supplemented with 10% FBS (Gibco BRL Co. New York, USA), penicillin (100 U/mL) and streptomycin (100 μg/mL). Both kinds of cells were cultured at 37 °C in a humidified atmosphere of 5% CO_2_.

Upon reaching 50–70% confluency, ECs were treated with normal concentration phosphate (0.9 mM of inorganic phosphate) or high concentration phosphate (3.5 mM of inorganic phosphate) (NaH_2_PO4:Na_2_HPO_4_ = 1:2, pH = 7.0) for 48 h. For transient transfection of miR-670-3p mimics, inhibitor or control oligos, a combination of oligos (50 nM) and Lipofectamine 3000 (Invitrogen, Carlsbad, USA) were mixed following the manufacturer’s instructions and added to cells in 6-well plates at a density of 2 × 10^5^ cells per well. MiR-670-3p mimics, inhibitor, and their control oligos were purchased from Ribobio (Guangzhou, China). VSMCs calcification was induced by incubation with 3.5 mM inorganic phosphate (Sigma-Aldrich,Saint Louis, USA). The ossification medium was subsequently changed every three days, and the cultured cells were treated with ECs-Exos (100 μg/mL) simultaneously. IGF-1 was knocked down in VSMCs by transient transfection of siRNA oligos (Ribobio, Guangzhou, China).

### Exosomes isolation, identification, and administration

In order to isolate exosomes, ECs receiving 0.9 mM Pi treatment or 3.5 mM Pi stimulation were cultured in the exosome-free medium for 48 h. Exosomes were isolated from ECs culture supernatants by the differential centrifugation method. Briefly, a total of 80 mL supernatant was centrifuged as follows: 300 × *g* for 10 min, 2000 × *g* for 30 min, 10,000 × *g* for 30 min, and finally ultracentrifugation at 110,000 × *g* for 90 min at 4 °C. Ultracentrifugation was accomplished by Optima XPN-100 UltraCentrifuge (Beckman, Germany) with SW 41 Ti rotor and 12.5 mL tube (Beckman, Germany). For isolation of plasma exosomes, the plasma sample was diluted by PBS buffer in a ratio of 1:4 before ultracentrifugation. By differential centrifugation, the nanoparticles were congregated at the bottom of the tube. After PBS washing and ultracentrifugation at 110,000 × *g* again, the exosomes-enriched pellet was re-suspended in PBS and processed through a 0.22 μm filter. Exosomes were concentrated by 10 kDa centrifugal filter units (Millipore, Billerica, USA) until the final volume was about 100 μL. The protein quantification of exosomes was performed using a BCA kit (Beyotime Biotechnology, Shanghai, China).

To identify the isolated exosomes, a Hitachi H-7650 transmission electron microscope (Hitachi, Tokyo, Japan) was used to observe the morphology of exosomes, and a molecular size analyzer (ZetaView PMX 110, Particle Metrix, Germany) was used to measure the size distribution of the exosomes. Western blot analysis was conducted to detect the expression of exosomal surface marker proteins CD9, CD81, and TSG101.

### Exosomes uptake analysis

To monitor the trafficking of exosomes, exosomes were labeled with a PKH26 Red fluorescent cell linker kit (MINI26-1KT, Sigma) according to the manufacturer’s instructions. Briefly, 5 µL of the PKH26 fluorescent probe was dissolved in 1 mL Diluent C solution and incubated with 100 µg exosomes at room temperature (RT) for 5 min. The staining step was terminated by 1 mL BSA. The labeled exosomes were precipitated by ultracentrifugation for 90 min at 110,000 × *g* after washing with PBS. The labeled exosomes were incubated with VSMCs at 37 °C, and 12 h later, VSMCs were fixed with 4% paraformaldehyde (PFA) for 20 min. Then, they were washed with PBS and stained with α smooth muscle actin (α-SMA) and DAPI, respectively. The effects of exosome uptake were observed under a laser scanning confocal microscope.

In mice, the stock solution of DiR (D12731, Thermo Fisher Scientific) was prepared in ethanol and a 300 mmol/L working solution was prepared (Sigma-Aldrich). Exosomes isolated from culture supernatant derived from ECs were incubated with 2 mmol/L DiR for 30 min. The exosomes were then washed with PBS and followed by ultracentrifugation for 90 min at 110,000 × *g* to remove free dye. The DiR dye alone or DiR-labeled exosomes (100 µg per mice) were injected into wild-type (WT) mice via the tail vein, and 12 h later, the mice were imaged and the thoracic aortas were collected. The aorta tissues were frozen in liquid nitrogen quickly to make frozen sections.

### Transwell co-culture experiments

Well inserts for six-well plates with a 0.4 μm pore-sized filter were purchased from Corning and used following the manufacturer’s instructions. ECs (1.5 × 10^5^) in the well inserts were pre-treated with GW4869 (Sigma-Aldrich) and cultured in a complete F12-K medium for 48 h. VSMCs (2 × 10^5^) were seeded into the six-well plate with DMEM. Before starting the co-culture experiments, both ECs and VSMCs were washed with PBS and then the insert ECs were put into the six-well plate with VSMCs. All co-culture experiments were done in complete DMEM supplement with HPi (3.5 mM Pi). After co-culture for 72 h, the protein of VSMCs were collected for further study.

### ALP activity

ALP activity was performed as previously described [18, 19]. Briefly, the VSMCs were washed with PBS and scraped into the solution. Spectrophotometric measurement of p-nitrophenol released at 37 °C was utilized to analyze ALP activity. ALP activity was normalized by the total protein content of the cell lysate.

### Alizarin Red S staining

Alizarin Red staining was performed as previously described [18, 19]. Briefly, for VSMCs, cells cultured with 100 μg/mL ECs-Exo for 14 days were fixed in 4% PFA and then stained with 0.2% (pH 4.2) Alizarin Red solution for 5 min at 37 °C. For artery samples, arteries were processed using the paraffin-embedded method, and the artery sections were stained with 0.2% (pH 4.2) Alizarin Red S solution for 1 min at RT. The stained matrixes or tissues were assessed and photographed using a digital microscope.

### RNA isolation and RNA analyses

Total RNA was extracted and purified from cells and exosomes using the miRNeasy® Mini Kit (Qiagen, cat. No. 217084) according to standard protocol. Briefly, 700 µL QIAzol lysis reagent was added, followed by chloroform extraction, and phase separation was achieved by centrifugation at 12,000 × *g* for 15 min at 4 °C. Total RNA was isolated from the aqueous phase by spin column purification. RNA concentration and purity were assessed using the RNA Nano Drop 2000 System (Agilent Technologies, CA, USA).

### miRNA microarray assay

To identify the differently expressed miRNAs in the exosomes released by ECs treated with 0.9 mM Pi or 3.5 mM Pi, miRNA microarray assays were performed with the help of Kangchen Biotech (Shanghai, China). The miRCURY™ Hy3™/Hy5™ Power labeling kit (Cat.No208032-A, Exiqon) was used according to the manufacturer’s guidelines for miRNA labeling. Briefly, 1μLRNA in 2.0 μL of water was mixed with 1.0 μL of CIP buffer and CIP (Exiqon) and incubated for 30 min at 37 °C. Then 3.0 μL of labeling buffer, 1.5 μL of fluorescent label (Hy3TM), 2.0 μL of DMSO, 2.0 μL of labeling enzyme were added into the mixture for incubation at 16 °C for 1 h. After stopping the labeling procedure, the Hy3™-labeled samples were hybridized on the miRCURYTM LNA Array (v.19.0) (Exiqon) according to the array manual. The total 25 μL mixture from Hy3™-labeled samples with 25 μL hybridization buffer were first denatured for 2 min at 95 °C, and incubated on ice for 2 min. Then hybridized to the microarray for 16–20 h at 56 °C in a 12-Bay Hybridization Systems (Hybridization System - Nimblegen Systems, Inc., Madison, WI, USA). After being washed several times using Wash buffer kit (Exiqon), the slides were scanned using the Axon GenePix 4000B microarray scanner (Axon Instruments, Foster City, CA).

Scanned images were then imported into GenePix Pro 6.0 software (Axon) for grid alignment and data extraction. Replicated miRNAs were averaged and miRNAs that intensities ⩾ 30 in all samples were chosen for calculating the normalization factor. Expressed data were normalized using the Median normalization. After normalization, significant differentially expressed miRNAs between two groups were identified through Fold change and *p*-value. Differentially expressed miRNAs between two samples were filtered through Fold change. Finally, hierarchical clustering was performed to show distinguishable miRNA expression profiling among samples.

### Quantitative real-time PCR (qPT-PCR)

Total RNA was extracted from cultured ECs, VSMCs, and ECs-Exos using the miRNeasy® Mini Kit (Qiagen, USA). For miR-670-3p, miR-155-3p, miR-148b-5p, and miR-185-3p analysis, the All-in-One™-miRNA-qRT-PCR detection system was used (AOMD-Q060, Genecopoiea) as described by the manufacturer’s protocol and using U6 snRNA as the reference. The detailed reaction conditions had been described in our previous study [19]. The PCR primers purchased from Genecopoiea were as follows: miR-670-3p (MmiRQP3302), miR-155-3p (MmiRQP3031), miR-148b-5p (MmiRQP3079) and miR-185-3p (MmiRQP3034) and U6 snRNA (MmiRQP9002). The relative standard curve method (2^-△△CT^) was used to determine the relative miRNA expression. The results were expressed as fold change relative to the relevant control.

### Digestion assay

Exosomes were digested by degrading enzymes and detergent. Firstly, a combined treatment of protease K (TIANGEN, China) and RNase A (TIANGEN, China) was added to the exosome preparation. Exosomes were treated with protease K (final concentration 100 µg/mL) for 30 min at 37 °C, then treated with RNase A (final concentration 100 µg/mL) for 15 min at 37 °C. On the other side, exosomal RNA was digested with Tritonx-100 (final concentration 10%) for 30 min at room temperature (RT), followed by RNase A (final concentration 100 µg/mL) for 15 min at 37 °C. The untreated aliquot was served as a control. Exosomal total RNA was extracted to evaluate the expression level of miR-670-3p by qRT-PCR.

### Western blot analysis

Protein expression was determined by western blot as previously described. In all, 30 μg protein was analyzed by SDS gel electrophoresis and then transferred to a polyvinylidene fluoride membrane. After blocking with 5% non-fat milk, the membrane was incubated with proper antibodies, including CD9(ab92726, 1: 1000, Abcam), CD81(ab21916, 1:500, Abcam), TSG101(ab125011, 1: 500, Abcam), IGF-1(20215-1-AP, Proteintech, 1:500) and Runx2 (ab23981, 1:1000, Abcam) at 4 °C overnight, then incubated with appropriate secondary antibody(1:4000 dilution) at RT for 1 h. Blots were processed using an enhanced chemiluminescence (ECL) kit and analyzed by Amersham Imager 600 (General Electric, USA).

### Plasmid constructs

To determine the function of miR-670-3p, a segment of the 3′ untranslated region (UTR) of human IGF-1 with the predicted binding sites of miR-670-3p was cloned into XbaI-FseI restriction sites of the pGL3 luciferase reporter vector (Promega, USA). The QuikChange site-directed mutagenesis kit (Stratagene, USA) was used to construct a mutant 3′ UTR of IGF-1.

### Luciferase reporter assay

VSMCs were co-transfected with a luciferase reporter carrying wild-type IGF-1 3′ UTR, mutant IGF-1 3′ UTR, and miR-670-3p mimics or scramble oligonucleotides. Then, 48 h after transfection, luciferase activities were quantified with the luciferase assay system (Promega, USA). The nucleotide sequences of primers for the construction and mutation of 3′ UTR IGF-1 mRNA were purchased from Ribobio (Guangzhou, China).

### Animals

The experimental C57BL/J mice (6- to 8-week old) were randomly divided into four different groups: sham operation, 5/6‐nephrectomy plus high‐phosphate diet‐treated (5/6 NTP), 5/6 NTP + DMSO, and 5/6 NTP + GW4869. The 5/6 NTP mouse model was set up successfully as described in our previous studies [18, 20] and sacrificed to dissect the thoracic aortas for further research. Immunohistochemistry analysis was used to test the expression of the Runx2 protein in aortic tissues. Alizarin Red S staining and Von Kossa staining were used to detect artery calcification. Artery calcium content was measured by the o‐cresolphthalein method. Total protein was quantified using the Bradford protein assay.

To generate endothelial-specific miR-670-3p transgenic mice, firstly, the miR-670-3p^flox/+^ mice and ROSA26-CAG-LSL-miR-670-3p^Tg/+^ mice were generated by CRISPR/Cas9 system with the help of Beijing Biocytogen Company (Beijing, China). In brief, for miR-670-3p^flox/+^ mice, the Cas9/guide RNA (gRNA) target sequences were designed for the regions upstream and downstream of miR-670. The targeting construct consisting of 1.4 kb arms of homologous genomic sequence immediately upstream (5′) and downstream (3′) of miR-670 flanked by two lox P sites. As for ROSA26-CAG-LSL-miR-670-3p KI mice, a cassette containing CAG promoter-loxP-Stop-loxP-MiR670-pA sequence was inserted at the Rosa26 locus (Fig. 2a) with homologous recombination. Finally, Cas9 mRNA, sgRNAs, and donor vector were injected into the cytoplasm of fertilized eggs. To further generate endothelial-specific miR-670-3p knock-out mice (miR-670-3p^EC-KO^ mice) and miR-670-3p knock-in mice (miR-670-3p^EC-KI^ mice), miR-670-3p^flox/+^ mice or mice ROSA26-CAG-LSL-miR-670-3p^Tg/+^ mice were bred to Tek-Cre mice (Strain Name: B6.Cg-Tg(Tek-cre)12Flv/J, The Jackson Laboratory), which specifically express Cre-recombinase under vascular endothelial-specific receptor tyrosine kinase promoters in vascular ECs. Genotyping of miR-670-3p^EC-KO^ and miR-670-3p^EC-KI^ mice was performed by PCR analysis with tail DNA according to a standard protocol, and the primer sequences used for genotyping were shown in Supplemental Table 1.

### Von Kossa staining

For Von Kossa staining, sections were incubated with silver nitrate (5%, Sigma) for 1 h under exposure to ultraviolet light, followed by incubation in sodium thiosulfate for 10 min. Photomicrographs were used for analysis by Image-Pro Plus software (version 6.0) to detect positive staining areas.

### Immunohistochemistry and immunofluorescence

Sections of the arteries isolated from mice were fixed and processed using the paraffin-embedded method or frozen in liquid nitrogen quickly to make frozen sections. Paraffin sections were deparaffinized in xylene and rehydrated in a graded ethanol series. Then, antigens were retrieved by trypsin and incubated with 3% hydrogen peroxide to clear endogenous peroxidase. After blocking with 5% BSA, slides were probed overnight at 4 °C with polyclonal antibodies against Runx2 (ab23981, 1:100, Abcam). The primary antibody was detected by a biotinylated secondary antibody, followed by the avidin-biotin-peroxidase complex and 3,3′-diaminobenzidine chromogen (catalog no. GK500710; Gene Tech, Shanghai). The immunopositive results were measured using a Nikon Eclipse microscope with a Nikon DSR1 camera and analyzed by Nikon NIS-Elements AR software (Nikon Instruments Korea, Seoul, Korea).

The frozen sections isolated from exosomes injected into mice were fixed with precooled PFA at 4 °C for 15 min and washed with PBS, followed by nuclear staining with DAPI (Invitrogen). The sections were incubated with an antibody mixture of CD81 and α-SMA at 4 °C overnight. After being washed with PBS-T buffer, the section was subjected to a mixture of secondary antibody incubation. Finally, the uptake of exosomes by VSMCs in the artery were observed under a fluorescence microscope (Nikon Instruments Korea, Seoul, Korea).

### Patient information and sample collection

A total of 15 patients with the diagnosis of CKD-5 at the Second Xiangya Hospital, Central South University, participated in this study. Healthy control volunteers were recruited from the physical examination center. Inclusion criteria and patient information was described in our previous work [21]. The blood sample was collected in an EDTA tube. After centrifugation at 2000 × *g* for 30 min at 4 °C, the plasma was aspirated and stored at −80 °C before use.

### Coronary artery calcification (CAC) measurement

CAC scores were calculated using the Siemens Somatom Definition computed tomography (CT) multilayer spiral scanner (Germany), and the calcification of coronary arteries was quantified via Agaston and analyzed by Siemens Ca Scoring software (syngo. via, Siemens Healthcare GmbH). A total CAC score was generated by using the Agatston method, which has been described in our previous work [19]. The measure of the area of calcification times a fixed coefficient (the maximum pixel density decision) and the total score of the calcification of all faults were termed as the CAC scores.

### Statistical analysis

The data were presented as mean ± standard deviation (SD), and the analysis was performed with GraphPad Prism software (GraphPad Prism version 6.0). The normality of data distribution was assessed before analysis, and Student’s *t*-test was used to compare normally distributed data between two different groups. Comparisons of multiple groups were made using a one-way analysis of variance (ANOVA). A level of *p* < 0.05 was considered statistically significant. All experiments were repeated at least three times, and representative experiment results are shown in the figures. Correlation analysis was performed using Spearman's *r* test(for abnormally distributed data).

## Results

### Culture media of ECs regulated VSMCs calcification

**Fig. 1:**
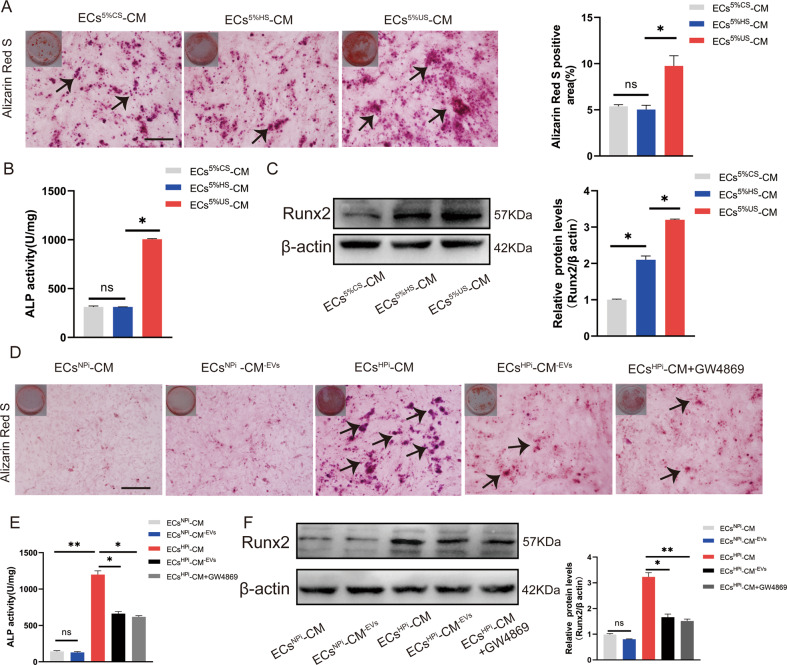
The effect of ECs culture media on VSMCs calcification. **A**–**C** 5% calf serum (CS), 5% healthy serum (HS), and 5% uremia serum (US) were supplemented to the culture media (CM) of mice ECs, respectively. These CM were collected for the following VSMCs cultures. Alizarin Red S staining (**A**), ALP activity (**B**), and Runx2 expression (**C**) were measured on day 14, day 7, and day 7, respectively. The black arrows indicate mineralized nodules in VSMCs. **D**–**F** VSMCs were cultured with 0.9 mM inorganic-induced endothelial CM (ECs^NPi^-CM), 3.5 mM inorganic phosphate-induced endothelial cells CM (ECs^HPi^-CM), and their EV-depleted conditional media (ECs^HPi^-CM^-EVs^, ECs^HPi^-CM^-EVs^, and ECs^HPi^-CM^-EVs^ + GW4869). Alizarin Red S staining (**D**), ALP activity (**E**), and Runx2 expression (**F**) were measured on day 14, day 7, and day 7, respectively (Scale bar = 100 μm). Three independent experiments were performed, and representative data were shown. Data are shown as mean ± SD. ns: no significance, *p < 0.05, **p < 0.01.

Arterial calcification is highly prevalent in patients with ESRD. Previous research and our data have shown that serum from uremia patients can promote osteogenic differentiation and calcification of VSMCs [22, 23] (Supplemental Fig. 1). It was revealed that both ECs and VSMCs play a fundamental role in the maintenance of vascular homeostasis. To investigate the role of ECs in arterial calcification under uremic conditions, 5% calf serum (CS), 5% healthy control serum (HS), and 5% uremia serum (US) were supplemented to complete the culture media of mice ECs. It was found that compared with HS, US can significantly promote the deposition of minerals, and the involvement of ECs could further accelerate the deposition of mineralized nodules induced by US (Supplemental Fig. 1). Alizarin Red S staining showed significantly vigorous intensity in VSMCs incubated with ECs culture media supplemented with US. Quantitative analysis is shown in the bar graph on the right (Fig. 1A). Moreover, the culture media of US-stimulated ECs also significantly promoted ALP activity and Runx2 expression (Fig. 1B, C). However, there was no significant change in the VSMCs incubated with healthy serum samples, suggesting the pro-calcifying effect of ECs exposed to US.

Hyperphosphatemia due to reduced renal phosphate clearance is commonly seen in US. The role of hyperphosphatemia was a significant risk factor in the development of vascular calcification. We collected the US with the same phosphate levels from three patients; surprisingly, the US induced Runx2 expression and ALP activity was not consistent, while the same concentration of inorganic phosphorus has better repeatability (Supplemental Fig. 2), since other uremia toxins, such as β2-microglobulin, indoxyl sulfate, homocysteine, uric acid, and parathyroid hormone may frustrate the pro-calcification effect of US [24]. In order to explore the appropriate phosphorus concentration, we cultured VSMCs with high phosphorus of 2.0, 2.8, 3.3, 3.5, and 4.0 mM respectively, and used Cell-Counting-Kit-8 to analyze the cell viability of 24, 48, and 72 h. Culturing VSMCs in a high-phosphate medium increased calcification in a dose-dependent manner, which peaked at 3.5 mM (Supplemental Fig. 3). Meanwhile, 3.5 mM high phosphorus did not affect the cell viability of ECs and VSMCs (Supplemental Fig. 4), So we chose 3.5 mM to simulate high phosphorus status in the subsequent investigations.

To determine the possible pro-calcifying substance of the culture media supplemented with 3.5 mM Pi in ECs (ECs^HPi^-CM), we depleted the extracellular vesicles (EVs) by ultracentrifugation in ECs culture medium or GW4869 pre-treatment with ECs. The 0.9 mM Pi-treated ECs culture media (ECs^NPi^-CM) served as a control. Amazingly, the removal of EVs by ultracentrifugation in ECs culture medium resulted in a significant reduction of the pro-calcifying potential of high phosphorus-induced media, as the calcium deposition, ALP activity, and Runx2 expression were decreased in the ECs^HPi^-CM^-EVs^ group (Fig. 1D–F). Meanwhile, pre-treatment of ECs with GW4869 to eliminate the formation and releasing of EVs also effectively reduced the pro-calcifying effect of high-phosphate treated ECs culture medium in VSMCs (Fig. 1D–F). Accordingly, neither complete ECs culture media stimulated by 0.9 mM Pi nor 0.9 mM Pi media with EVs depletion had an influence on VSMCs calcification. Taken together, all the results implied that EVs played a crucial role in the crosstalk between ECs and VSMCs under uremic/high-phosphate conditions.

### Exosomes isolated from high phosphorus-treated ECs culture media could be uptaken by VSMCs and promoted VSMCs calcification

**Fig. 2:**
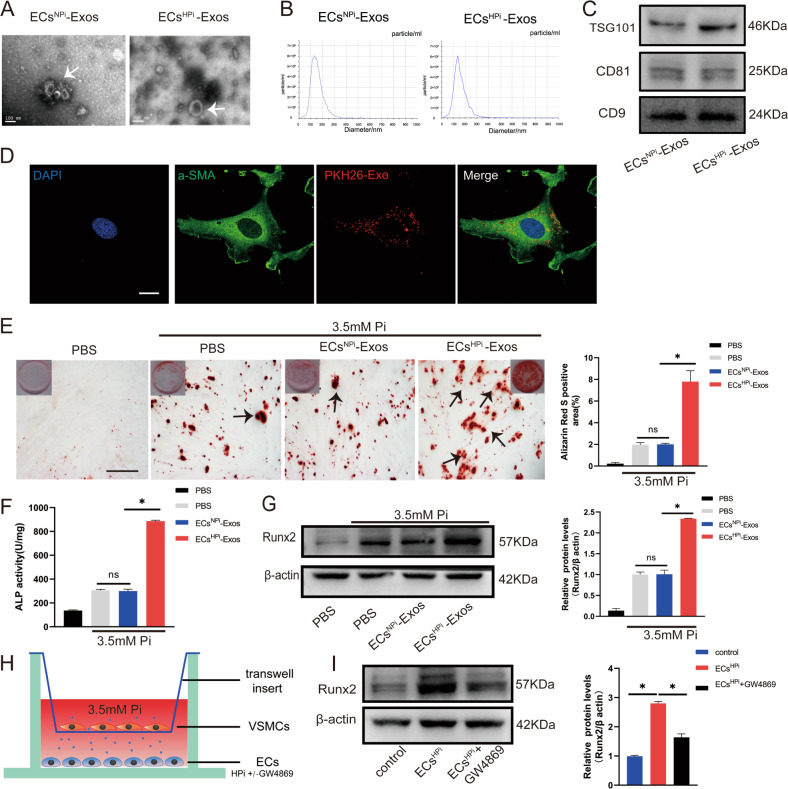
Exosomes secreted from high phosphorus-stimulated ECs exacerbated VSMCs calcification. **A** Transmission electron micrographs of exosomes derived from ECs^NPi^-Exos or ECs^HPi^-Exos phosphate conditions (Bar = 100 nm). **B** The nanoparticle concentration and size distribution of the ECs-Exos. **C** CD9, CD81, and TSG101 immunoblots of exosomes. **D** VSMCs were incubated with PKH26 fluorescently labeled exosomes for 12 h. Confocal microscopy analysis was used to identify the uptake of EVs by VSMCs (PKH26 in red, DAPI in blue, and α-SMA in green) (Scale bar = 5 μm). **E** VSMCs were incubated with ECs ^NPi^-Exos and ECs^HPi^-Exos for 48 h, respectively. Then, Alizarin Red S staining was performed and calcium content was measured in VSMCs. The black arrows indicate mineralized nodules in VSMCs (Scale bar = 200 μm). **F** ALP activity was measured by an ALP kit in VSMCs incubated with ECs^NPi^-Exos and ECs^HPi^-Exos. **G** The expression of Runx2 was determined by Western blot in VSMCs incubated with ECs^NPi^-Exos and ECs^HPi^-Exos. **H**, **I** ECs in the lower chamber pre-treated with or without GW4869 were co-cultured with VSMCs in six-well Transwell units, and the upper chamber VSMCs were harvested to determine the expression of Runx2 by using western blot. Three independent experiments were performed, and representative data were shown. Data are shown as mean ± SD. ns: no significance, *p < 0.05.

We isolated the exosomes from ECs culture media to investigate the role of exosomes in VSMCs calcification. Firstly, to identify whether the isolated extracellular vesicles from the supernatant of ECs treated with high phosphorous (3.5 mmol/L) were exosomes, transmission electron microscopy was utilized and identified that the vesicles were round-shaped with a double-layered membrane structure (Fig. 2A). Nanoparticle Tracking Analysis (NTA) revealed ECs^NPi^ Exo and ECs^HPi^ Exo had a mean diameter of vesicles was 127.3 ± 42.2 and 115.5 ± 58.3, respectively, which exhibited a typical single sharp peak (Fig. 2B). The exosome protein yield as well as particle numbers per milliliter was significantly higher in the HPi group (ECs^HPi^ Exo) compared to NPi treated group (ECs^NPi^ Exo) (Supplemental Fig. 5A, B). There was no significant difference between the number of particles isolated per milliliter as well as the amount of particles per 100 μg protein between the two groups (Supplemental Fig. 5C). Western blot further showed the vesicles were positive for the surface marker proteins CD9 and CD81, as well as tumor susceptibility gene 101 (TSG101) (Fig. 2C). Besides, the characters of vesicles isolated from ECs treated with 0.9 mM Pi were similar to that of 3.5 mM induced ECs (Fig. 2A–C). Together, these three features suggested that the types of EVs purified from the supernatant of ECs were consistent with the characteristics of exosomes.

Considering that arterial calcification mainly occurs in VSMCs of the arterial media wall in patients with ESRD, we wonder whether the exosomes released by ECs participate in the regulation of VSMCs calcification. Firstly, to identify whether the exosomes derived from ECs could be uptaken by the VSMCs, the exosomes were labeled with red fluorescent dye PKH26 and then incubated with VSMCs. Laser confocal scanning electron microscopy revealed that VSMCs exhibited efficient uptake of ECs-derived exosomes, as evidenced by the red fluorescence incorporated into VSMCs (Fig. 2D). Furthermore, the effects of high concentrations of ECs^HPi^-Exos on VSMCs calcification were evaluated. Alizarin Red S staining showed that mineralized nodules were greatly increased in ECs^HPi^-Exos-induced VSMCs for 14 days (Fig. 2E). We also found that the levels of ALP activity and Runx2 protein were significantly increased in ECs^HPi^-Exos-induced VSMCs when compared with VSMCs treated with exosomes derived from ECs ^NPi^ -Exos (Fig. 2F, G).

Besides, in order to explore whether exosomes were involved in cell-cell communication between ECs and VSMCs, a transwell co-culture assay of ECs with VSMCs in corning chambers was carried out, in which cells were physically separated by a membrane with 0.4 µm pore size to prevent direct cell-cell contact but allow exosomes to exchange freely (Fig. 2H). We found that, when VSMCs co-cultured with ECs which were pre-treated with 3.5 mM Pi, the level of Runx2 protein in VSMCs was increased significantly (Fig. 2I). However, when ECs were incubated with GW4869 prior to co-culture with VSMCs, the high phosphorus-induced Runx2 expression in VSMCs was blocked significantly(Fig. 2I), which suggesting a critical role of exosomes.

Taken together, these results suggested that it was the exosomes, rather than other factors, in the supernatant from high phosphorus-induced ECs that promoted VSMCs calcification in vitro.

### miR-670-3p was the major function molecular in endothelial exosomes induced by high phosphorus

**Fig. 3:**
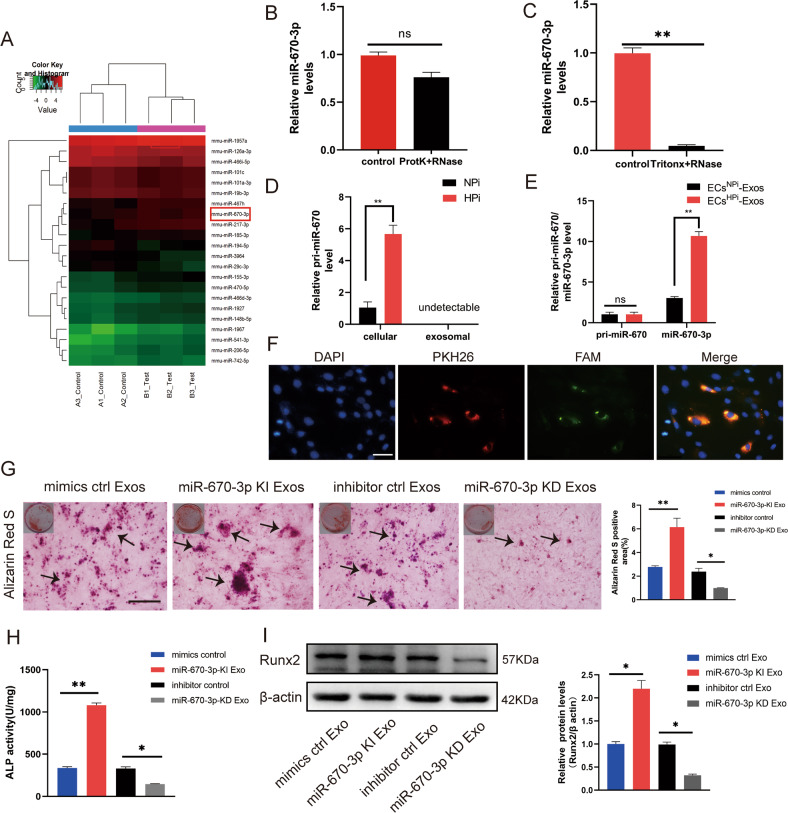
miR-670-3p was enriched in ECs^HPi^-Exos and regulated VSMCs calcification. **A** The differentially expressed miRNAs (a cut-off of absolute fold change ≥1.5 and *p* < 0.05) between ECs^NPi^-Exos and ECs^HPi^-Exos according to microarray analysis. **B** qRT-PCR quantitative results of miRNA after exosomes were treated with proteinase K and RNase. **C** qRT-PCR quantitative results of miRNA after exosomes were treated with Tritonx-100 and RNase. **D** Expression levels of pri- miR-670-3p in ECs and EC-Exos. **E** Expression levels of pri-miR-670-3p and mature miR-670-3p in VSMCs. **F** Confocal microscopy analysis was used to verify whether exosomal miR-670-3p could be uptaken by VSMCs. VSMCs were cultured with PKH26-labeled exosomes derived from ECs transfected FAM-miR-670-3p-mimics. The FAM-miR-670-3p signals were detected in the cytoplasm of VSMCs (Green), and FAM-miR- 670-3p signals were co-localized with PKH26 in VSMCs (PKH26 in red and DAPI in blue), (scale bar = 50 μm). **G**–**I** VSMCs were incubated with miR-670-3p knocked-in exosomes or miR-670-3p knocked-down exosomes during a high phosphorus induction period. Then, the severity of VSMC calcification was evaluated by Alizarin Red S staining (**G**), ALP activity (**H**), and Runx2 protein expression (**I**). The black arrows (**G**) indicate mineralized nodules in VSMCs (scale bar = 100 μm). Three independent experiments were performed, and representative data were shown. Data were shown as mean ± SD. ns: no significance, *p < 0.05, **p < 0.01. ns: no significance.

Previous studies have demonstrated exosomes regulated a large number of physiological activities via non-coding RNAs, including miRNAs with multiple functional properties. Thus, we hypothesized that ECs-derived exosomes are involved in regulating VSMCs calcification through transferring specific miRNA. Hence, a microarray-based miRNA expression profiling analysis was conducted to identify the differentially abundant miRNAs between ECs^NPi^-Exos and ECs^HPi^-Exos (Fig. 3A). Among them, 161 significantly upregulated miRNAs and 82 significantly downregulated miRNAs in ECs^HPi^-Exos were identified (fold change ≥ 1.5 and *p* ≤ 0.05). qRT-PCR confirmed that the expression level of miR-670-3p, as well as miR-155-3p, miR-185-3p, and miR-148b-5p, was dramatically increased in ECs^HPi^-Exos compared with that of ECs^NPi^-Exos (Supplemental Fig. 6A).

In order to test whether miR-670-3p, miR-185-3p, miR-155-3p and/or miR-148b-5p participate in regulating VSMCs calcification, VSMCs were transfected with miR-670-3p, miR-185-3p, miR-155-3p and miR-148b-5p mimics or miRNA scramble control (miRNA NC). NC is a validated negative control that has no homology with the target gene sequence. The results showed that only overexpressing miR-670-3p could increase the expression of Runx2 in VSMCs (Supplemental Fig. 6B), indicating miR-185-3p, miR-155-3p and miR-148b-5p did not have major functions in ECs^HPi^-Exos in arterial calcification. Therefore, we chose miR-670-3p enriched in ECs^HPi^-Exos for further study. Furthermore, to ensure that the miRNA originated from exosomes instead of free RNA-binding protein, exosomes were digested with proteinase K and RNase before RNA extraction. The miR-670-3p level was measured by qRT-PCR. Results showed that miR-670-3p in ECs^HPi^-Exos exhibited resistance to proteinase K and RNase digestion because of the protection of the lipid membrane (Fig. 3B). Furthermore, upon adding a detergent (Triton-X) to dissolve the lipid bilayer of the exosomes, treatment with RNase was able to eliminate the miR-670-3p cargo in exosomes (Fig. 3C).

To further confirm that miR-670-3p can be transferred from ECs to VSMCs via exosomes, we detected the primary (pri)-miR-670 and mature miR-670-3p levels in ECs treated with 0.9 mM Pi or 3.5 mM Pi and their subsequently secreted exosomes. We found that pri-miR-670 only exhibited higher intracellular levels in ECs incubated with 3.5 mM Pi, while it was undetectable in both ECs^NPi^-Exos and ECs^HPi^-Exos (Fig. 3D). Furthermore, an increase in cellular levels of mature miR-670-3p, but not pri-miR-670, was observed in recipient VSMCs following their incubation with ECs^HPi^-Exos (Fig. 3E). These data suggested that the increased mature miR-670-3p in VSMCs were transferred from high phosphorus-induced ECs but not synthesized by themselves. Moreover, to investigate whether the miR-670-3p was transferred from ECs into VSMCs via exosomes, we first transfected ECs with green fluorescence-labeled FAM-miR-670-3p oligo. We then labeled the secreted exosomes containing FAM-miR-670-3p with PKH26 (red fluorescence). The PKH26-labeled exosomes containing FAM-miR-670-3p were cultured with VSMCs for 24 h. As expected, fluorescence imaging of VSMCs showed that both the red and green signals could be detected in the cytoplasm of VSMCs in the co-culture system (Fig. 3F), which demonstrated that VSMCs could be uptaken exosomes containing miR-670-3p secreted by high phosphorus-induced ECs.

To ensure the effect of ECs-Exos on VSMCs calcification was mediated by carrying miR-670-3p, we upregulated or downregulated the expression level of miR-670-3p in ECs-Exos by transfecting miR-670-3p mimics or inhibitor, respectively. Data showed the miR-670-3p level was increased significantly in exosomes transfected with miR-670-3p mimics (miR-670-3p KI Exo). In contrast, the level of miR-670-3p was significantly decreased in exosomes transfected with miR-670-3p inhibitor (miR-670-3p KD Exos) compared to those transfected with the miRNA control (inhibitor control Exos) (Supplemental Fig. 6C). As expected, the formation of calcium nodules was significantly increased in VSMCs treated with miR-670-3p KI Exos, while they were markedly decreased in VSMCs treated with miR-670-3p KD Exos (Fig. 3G). Similar results were seen in the ALP activity and Runx2 protein (Fig. 3H, I).

These results demonstrated ECs original exosomal miR-670-3p played a vital role in the pro-calcification effect in VSMCs.

### IGF-1 was the target gene of miR-670-3p and involved in regulating VSMCs calcification

**Fig. 4:**
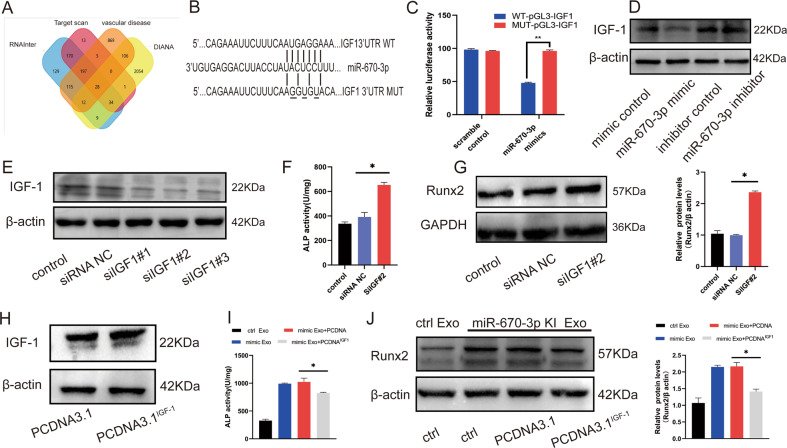
IGF-1 was the direct target gene of miR-670-3p and regulated VSMCs calcification. **A** Venn diagram of the predicted target gene of miR-670-3p from Target scan (red), RNAInter (blue), DIANA (yellow), and vascular calcification disease (orange). Three genes were identified as miR-670-3p target genes. **B** Schematic representation of miR-670-3p putative target sites in IGF-1 3′-UTR and alignment of miR-670-3p with WT and MUT IGF-1 3′-UTR showing pairing. **C** Luciferase reporter assays were performed using luciferase constructs carrying a wild type or mutant IGF-1 3′-UTR co-transfected into VSMCs with miR-670-3p mimics compared with empty vector control. Firefly luciferase activity was normalized to renilla luciferase activity. **D** The expression level of IGF-1 protein in VSMCs transfected with miR-670-3p mimics or miR-670-3p inhibitor was indicated by western blot. **E** The efficiency of IGF-1 knockdown in VSMCs by siRNA was measured by western blot. **F** ALP activity was measured in the VSMCs treated with siIGF^#2^ or siRNA control. **G** Runx2 expression was measured in the VSMCs treated with siIGF^#2^ or siRNA control. **H** The expression level of IGF-1 protein in VSMCs transfected with plasmid control or IGF-1 plasmid was indicated by western blot. **I**, **J** IGF-1 overexpressed plasmid and miR-670-3p mimics were transfected into VSMCs, then ALP activity (**I**) and Runx2 expression (**J**) were measured in the VSMCs. Three independent experiments were performed, and representative data were shown. Data were shown as mean ± SD. *p < 0.05.

miRNAs usually exert their functions by interacting with the 3′untranslated region (3′UTR) or protein-coding sequence of target mRNAs. To search for the downstream mechanism of miR-670-3p in regulating VSMCs calcification, bioinformatics software, including TargetScan, PicTar, and miRanda, were used to predict the possible targets of miR-670-3p. Interestingly, IGF-1 was chosen as the candidate gene because miR-670-3p was predicted to have a potential binding site in the 3′ UTR of IGF-1 (Fig. 4A, B), and IGF-1 was reported to play a crucial role in regulating osteoblast differentiation and osteogenesis [25–28]. IGF-1 (25 ng/mL) significantly protected VSMCs from osteogenic differentiation and mineral deposition [29]. To test whether exosomal miR-670-3p directly targets IGF-1 in VSMCs, a luciferase reporter plasmid containing the wild-type 3′ UTR of IGF-1 (pGL3-IGF-1 WT‑3′ UTR) was generated. When VSMCs transfected with pGL3-IGF-1 WT‑3′ UTR or pGL3-IGF-1 Mut‑3′ UTR reporter plasmids were incubated with miR-670-3p mimics, the luciferase activity of pGL3-IGF-1WT‑3′ UTR was significantly reduced. However, this inhibitory effect was largely blocked after the nucleotides in the putative binding site of miR-670-3p were mutated (Fig. 4C). This result suggested that IGF-1 might be the downstream effector of miR-670-3p in VSMCs.

Moreover, we measured the expression of IGF-1 protein in VSMCs after being transfected with miR-670-3p mimics or miR-670-3p inhibitors. The overexpression of miR-670-3p significantly decreased the level of IGF-1 protein in VSMCs, whereas miR-670-3p inhibitor transfection moderately increased the expression level of IGF-1 in VSMCs (Fig. 4D). These data further verified that IGF-1 was the direct target of miR-670-3p.

Furthermore, to explore the role of IGF-1 in miR-670-3p modulated VSMCs calcification, experiments were conducted in VSMCs in which IGF-1 small interfering RNA (siIGF-1) were transfected to knock down the expression of IGF-1. Western blot analysis verified that transfection of VSMCs with siIGF-1 successfully reduced IGF-1 protein expression (Fig. 4E). What’s more, knocking down IGF-1 led to increased ALP activity (Fig. 4F) and Runx2 protein expression (Fig. 4G). Given that high levels of miR-670-3p also result in increased calcification, we constructed an IGF-1 overexpression plasmid using PCDNA3.1 vector and transfected it into VSMCs (Fig. 4H). We found that the IGF-1 overexpression could partially abolish the pro-calcification effect of miR-670-3p overloaded exosomes (Fig. 4I,J). These experiments verified that IGF-1 was the downstream target of endothelial-original miR-670-3p and was involved in regulating VSMCs calcification.

### ECs-Exos could be internalized by the artery and mediated arterial calcification

**Fig. 5:**
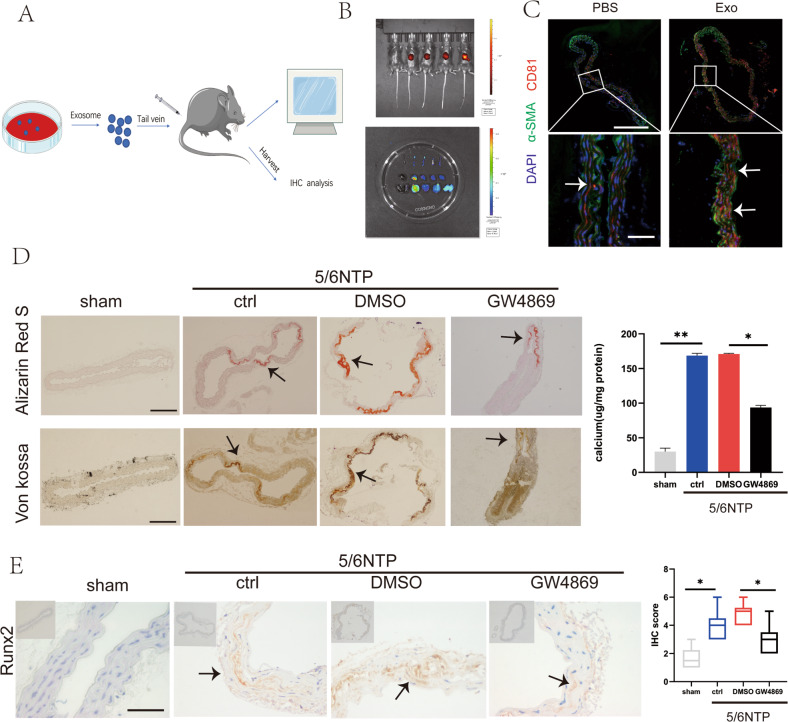
ECs-Exos could be internalized by the artery and mediated arterial calcification. **A** Schematic representation of procedure to trace ECs-Exos in mice. The ECs-Exos were labeled with the membrane-philic dye DIR and injected into mice through the tail vein. PBS was used as a negative control, and DIR dye was used as a positive control (*n* = 5 per group). **B** After 24 h, fluorescence signals were detected in the living mice (upper panel) and their organs after execution (lower panel). **C** The thoracic aorta was obtained to analyze the uptake of endothelial exosomes in the slices. Blue fluorescence (DAPI)-labeled cell nuclei, green fluorescence (Alexa 488)-labeled α -SMA, and red fluorescence (Alexa 555)-labeled CD81 indicating exosomes(The upper scale bar = 200 μm, the lower scale bar = 20 μm). The white arrow indicates CD81 positive exosomes. **D**, **E** Evaluation of the effect of pre-treatment of the exosome blocker GW4869 on arterial calcification induced by 5/6 nephrectomy. **D** Alizarin Red S (upper channel) and Von Kossa (lower channel) staining analysis of paraffin-embedded mouse vascular tissues. The arrows indicate mineralized nodules (scale bar = 200 μm). **E** Immunochemistry analysis of osteogenic differentiation marker Runx2 expression. The arrows indicate the positive expression of Runx2 in the mouse artery (scale bar = 50 μm). Data are represented as the mean ± SD, with five replicates for each group. Two-way ANOVA analyzed significance with Turkey’s HSD post hoc analysis. *p < 0.05, **p < 0.01.

Previous work suggested that exosomes could be uptaken by a variety of organs in vivo, such as bone and heart. In order to further clarify the role of exosomes, we performed in vivo experiments to directly test whether exosomes released from ECs could be uptaken by the vascular wall and played a role in arterial calcification. We labeled ECs-derived exosomes with the near-infrared dye DiR and injected them into wild-type mice via the tail vein. An experimental schematic was shown in Fig. 5A. Mice photography mainly detected the fluorescence signal in the liver (Fig. 5B). Considering that the relatively stronger fluorescence signal of the liver masked the fluorescence signals of other organs, we dissected the mouse, removed the liver and repeated the imaging. Photographs showed that the fluorescent signals of the DiR-labeled exosomes entered the aorta, kidney, and spleen within 12 h after injection in vivo (Fig. 5B), and immunofluorescence staining further confirmed that the exosomes marker CD81 was observed in the cytosol of mouse VSMCs after being injected with ECs exosomes (Fig. 5C).

5/6 NTP mice was known as an uremic arterial calcification model. By Von Kossa staining and Alizarin Red S staining, increased calcification in control 5/6 NTP mice compared with sham operation was observed. Interestingly, when GW4869 was used, a pharmacological compound known to effectively inhibit exosomes biogenesis/release in vivo, the effect of 5/6 NTP on promoting arterial calcification was partially blocked (Fig. 5D). Furthermore, our data demonstrated that the expression intensity of Runx2 of the mice aorta from the GW4869 pre-treatment group was much lower compared with other groups with immunochemistry analysis (Fig. 5E). Therefore, it was truly the endogenous exosomes that participated in arterial calcification regulation under uremic conditions.

### miR-670-3p regulated artery calcification in mice

**Fig. 6:**
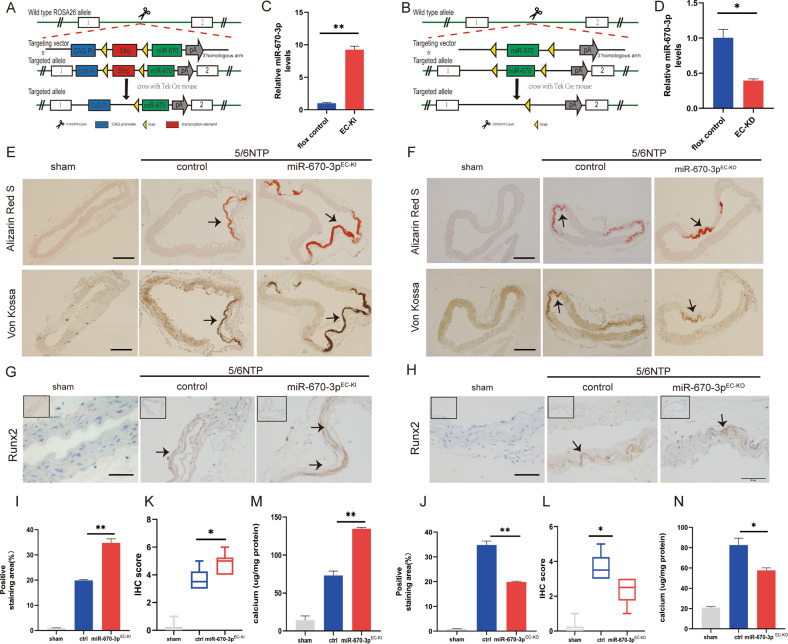
miR-670-3p regulated artery calcification in mice. We generated miR-670-3p endothelial cell-specific knock-in (miR-670-3p^EC-KI^) and knock-out (miR-670-3p^EC-KO^) mice, and animals were randomized into several groups with different treatments (*n* = 5 per group). **A**, **B** Schematic diagrams of the development strategy of the miR-670-3p^EC-KI^ and miR-670-3p^EC-KO^ mice. **C**, **D** The level of miR-670-3p in the aortas of miR-670-3p^EC-KI^ and miR-214-3p^EC-KO^ mice was measured by qRT-PCR, respectively. **E**, **F** The formation of arterial calcification was measured in miR-670-3p^EC-KI^ and miR-670-3p^EC-KO^ mice, respectively. The upper panel was stained with the Alizarin Red S. The lower panel was stained with the von Kossa. The arrows indicate mineralized arteries (scale bar = 200 μm). **G**, **H** Runx2 expression was evaluated with immunohistochemistry staining in miR-670-3p^EC-KI^ and miR-670-3p^EC-KO^ mice, respectively. The arrows indicate the positive expression of Runx2 in the mouse artery (Scale bar = 50 µm). **K**, **L** The IHC score of Runx2 staining in figure **G** and figure **H** was analyzed. **I**, **J** The quantification results of mineralized nodules staining in figure **E** and figure **F**. **M**, **N** The calcium content of the thoracic aorta of mice was measured by the o-cresolphthalein method. Results were represented by mean ± SD, with five replicates for each group. Two-way ANOVA analyzed significance with Turkey’s HSD post hoc analysis. *p < 0.05, **p < 0.01.

To analyze the role of miR-670-3p in regulating artery calcification in vivo. We generated a mouse strain containing the miR-670-3p knock-in or knock-out allele (Fig. 6A, B). The TEK mouse was an important tool mouse with cre activity for endothelial cells to specifically induce LoxP recombination, which could tissue-specific delete of gene fragments between two LoxP sites. We identified genotype with PCR amplification of tail genomic DNA (Supplemental Fig. 7C–E).

While miR-670-3p^EC-KO^ and miR-670-3p^EC-KI^ mice were born at the expected Mendelian ratio and had no gross abnormalities compared with wild-type mice (Supplemental Fig. 7A, B). To demonstrate that miR-670-3p was knocked in or knocked out in an endothelial cell-specific manner, aortic tissues and other organs of miR-670-3p^EC-KI^ and miR-670-3p^EC-KO^ mice were prepared and analyzed by qRT-PCR. As shown in Fig. 6C, D and Supplemental Fig. 7F, G, the expression of miR-670-3p were especially increased in artery tissues of miR-670-3p^EC-KI^ mice, while miR-670-3p expression was especially deficient in artery tissues of miR-670-3p^EC-KO^ mice when compared with that of floxed littermates.

To investigate the role of miR-670-3p in arterial calcification in vivo, 5/6 NTP mice was used as an arterial calcification model. The area and degree of artery calcification in the sham operation, 5/6 NTP, miR-670-3p^EC-KI^ + 5/6 NTP and miR-670-3p^EC-KO^ + 5/6 NTP mice were measured by Alizarin Red S staining and Von Kossa staining. The results showed that aortas derived from 5/6 NTP mice displayed extensive calcification compared with those from the sham operation group of mice,. In contrast, the calcification area was significantly increased in the miR-670-3p^EC-KI^ + 5/6 NTP mice but decreased in the miR-670-3p^EC-KO^ + 5/6 NTP mice (Fig. 6E, F). Figure 6I, J were the quantification of mineralized nodules staining. In the meantime, the results of the osteoblastic differentiation marker Runx2 were similar to those of Alizarin Red S staining (Fig. 6G, H). The quantification of Runx2 staining was in Fig. 6K, L. Moreover, the calcium deposited in the aorta of the mice was also detected to be increased in miR-670-3p^EC-KI^ + 5/6 NTP mice but decreased in the miR-670-3p^EC-KO^ + 5/6 NTP mice (Fig. 6M, N); all these results showed that miR-670-3p could promote artery calcification in mice.

### Elevated circulating exosomal miR-670-3p associated with reduced IGF-1 in patients with ESRD

**Fig. 7:**
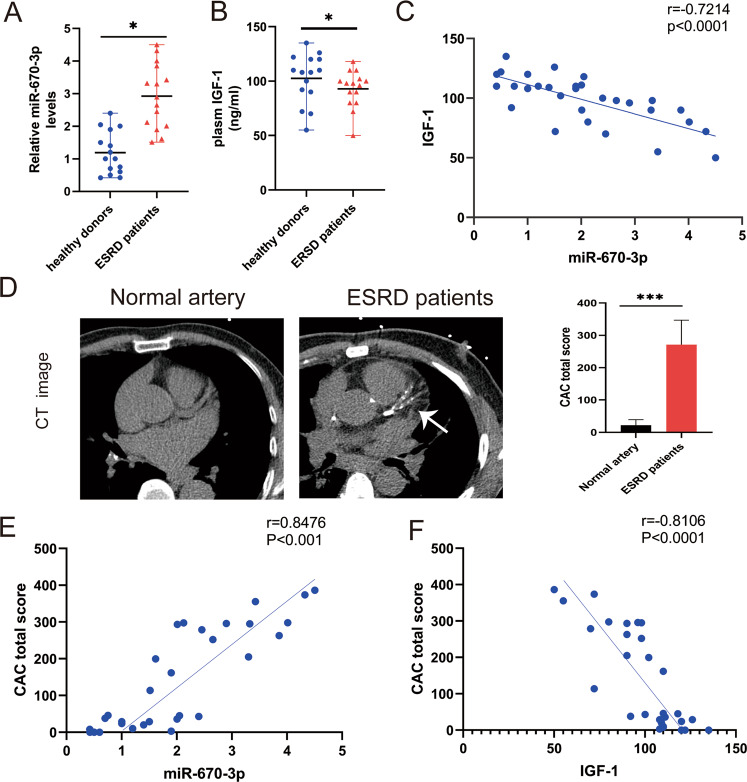
Circulating exosomal miR-670-3p was correlated with artery calcification in patients with ESRD. **A** qRT-PCR detection of miR-670-3p levels in plasma exosomes of healthy controls and patients with ESRD (*n* = 15). **B** The level of IGF-1 in the plasma of healthy people and patients with ESRD was measured by ELISA (*n* = 15). **C** Analysis of linear correlation between exosomal miR-670-3p and IGF-1 in plasma. **D** Analysis of coronary vascular calcification scores of normal people and patients with ESRD by multilayer spiral CT reconstruction, and representative pictures were shown (*n* = 15, left panel). The quantification results of CAC score in normal people and patients with ESRD (right panel). **E** Analysis of Spearman correlation between exosomal miR-670-3p and CAC score. **F** Analysis of Spearman correlation between plasma IGF-1 and CAC score. One-way ANOVA with Bonferroni’s post hoc test (**A**, **B**) and Spearman’s *r* test (**C**, **E**, **F**) were used. Data represented the mean ± SD. *p < 0.05.

Finally, we verified the regulatory relationship between miR-670-3p and IGF-1 in patients with ESRD versus age- and sex-matched healthy volunteers (Supplemental Table 2). Plasma exosomal miR-670-3p levels were significantly higher in patients with ESRD as compared with those in matched healthy controls (Fig. 7A). This upregulation was accompanied by a significant reduction in circulating IGF-1 levels (Fig. 7B). Furthermore, a negative correlation between plasma exosomal miR-670-3p and plasma IGF-1 was identified (Fig. 7C). The CAC score was also significantly higher in the ESRD group compared to that in healthy controls (Fig. 7D). As expected, a solid correlation was identified between plasma exosomal miR-670-3p and CAC total score (Fig. 7E), and the CAC total score was inversely correlated with circulating IGF-1 (Fig. 7F). In summary, these data showed that the increased plasma exosomal miR-670-3p and decreased IGF-1 were associated with media arterial calcification in patients with ESRD, indicating their potential as disease predictors.

## Discussion

**Fig. 8:**
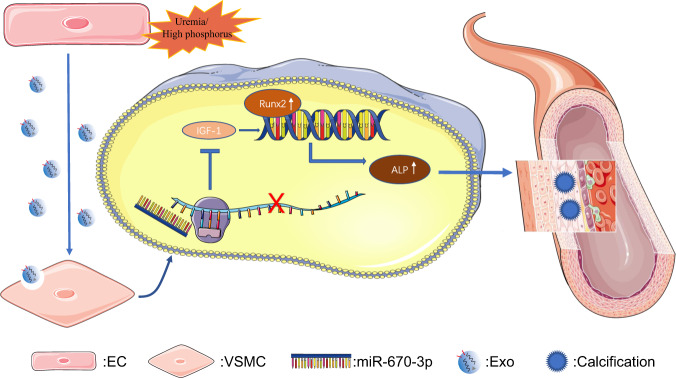
The mechanism diagram about arterial calcification mediated by crosstalk between ECs and VSMCs. Under uremia/high phosphorous conditions, ECs released exosomal miR-670-3p, promoting the expression of Runx2 and subsequently ALP to accelerate VSMC calcification, which was further accomplished by downregulating IGF-1. Finally, the accumulation of VSMCs calcification resulted in calcified arteries.

In the present study, we showed that exosomes released by ECs under uremic/high-phosphate conditions promoted media arterial calcification. Furthermore, miR-670-3p, enriched in ECs^HPi^-Exos, mediated the pro-calcifying effect. Meanwhile, IGF-1 was the target gene of miR-670-3p to regulate the osteogenic differentiation of VSMCs. Of note, we demonstrated that exosomal miR-670-3p derived from ECs regulated VSMCs calcification both in vitro and in vivo (Fig. 8). This analysis revealed a novel finding that had extended knowledge of the molecular mechanism underlying arterial calcification.

Arterial calcification often coincides in patients with type 2 diabetes, CKD, ESRD, aging, and other less frequent disorders [3, 30]. Medial artery calcification was a robust independent predictor of mortality in patients with type 2 diabetes mellitus, and it was also a significant predictor of future cardiovascular disease (CVD) events (including fatal or nonfatal myocardial infarction), stroke, and amputation [1, 31]. CAC predicts the risk of CVD in patients with CKD [32, 33]. Initially, arterial calcification was thought to result from passive degenerative processes. It was well-acknowledged that arterial calcification was an active biological process similar to osteogenesis [2]. Recent studies have illustrated that arterial calcification is a regulated process whose critical step was the trans-differentiation of VSMCs into osteoblast-like cells. A big challenge presents in this field, as no effective therapies for arterial calcification are currently available [34]. Thus, arterial calcification has become the focus of research by an increasing number of cardiovascular investigators.

For decades, researchers studying arterial calcification focused mainly on the trans-differentiation of VSMCs into osteochondrogenic cells [17–21, 35, 36] and, to a great extent, neglected the crucial role of ECs. The internal surface of the vasculature consists of a single cell layer of heterogeneous ECs (intimal layer). The strategic positioning of the endothelium allows it to sense pathological triggers circulating in the blood (mineral imbalance, uremic toxins, inflammation, etc.) and adequately respond to these hemodynamic changes. As such, the endothelium plays a crucial role in maintaining vascular homeostasis, maintaining a balance between endothelium-derived relaxing and contracting factors [4]. The physiological functioning of VSMCs in the arterial medial layer greatly depends on normal ECs behavior. Altogether, endothelial dysfunction is the initial step of major CVD under certain pathologic conditions [37]. It is reasonable to speculate that the dysfunction of ECs was a helping hand for VSMCs calcification. In rat models with spontaneous hypertensive, ECs have the ability to promote smooth muscle cell calcification [38]. High glucose-stimulated ECs also enhanced VSMCs calcification [39]. HUVECs exposed to phosphate and indoxyl sulfate accelerate arterial calcification by inhibiting the calcification inhibitor osteopontin (OPN) [40]. Generally, the normal serum phosphorus fluctuates between 0.8 and 1.6 mM, while patients with ESRD have much higher blood phosphorus levels. According to previous research [18, 19, 41], we chose 3.5 mM as high phosphorus and 0.9 mM as normal phosphorus concentration control for our exploration. In our present research, ECs respond to high-phosphate stimuli, thus secreting exosomes to transmit microRNAs to VSMCs and activate important molecular pathways involved in arterial calcification. The communication between ECs and VSMCs might provide novel insights into the molecular mechanisms underpinning arterial calcification. This process might be the driving force in the vicious circle of arterial calcification.

Exosomes, secreted by many kinds of cells, transfer cargo, such as lipids, proteins, and microRNAs, from the donor cells to recipient cells, thereby affecting the target’s function. Novel discoveries related to EVs, microRNAs, and calciprotein particles continue to reveal the mechanisms that are involved in the initiation and progression of arterial calcification [42, 43]. Matrix vesicles from calcifying VSMCs could accelerate calcification by inducing cell signaling changes and phenotypic alteration of recipient VSMCs [44]. Some factors, such as environmental calcium stress, increased the quantity of calcifying exosomes secreted from VSMCs, thereby promoting arterial calcification [45]. However, there is scant research designed to investigate the role of exosomes from intimal ECs on media arterial calcification. Early research showed that, in platelet-free plasma from patients with CKD, there was an increase in CD 31+/Annexin V+endothelial microparticles (EMPs) accompanied by a decrease in the number of endothelial progenitor cells (EPCs) [37]. Endothelial microparticles also mediated inflammation-induced arterial calcification. The CD 31^+^/Annexin V^+^EMPs in HUVEC cultures from TNF-α-stimulated ECs contained a significant amount of BMP-2 and were capable of enhancing VSMC osteogenesis and calcification [5]. These results confirmed the release of osteogenesis particles from ECs when they are exposed to harmful situations. Nowadays, the verification of exosomes makes it easier for researchers to trace the information transferred between cells. We found that the PKH26-labeled endothelial exosomes could be uptaken by VSMCs. What’s more, the endothelial exosomes could target the cardiovascular system, which was revealed by the organ-specific fluorescent signals detected within the mice. Our research offered direct evidence for the excretion of calcifying exosomes in the arterial intima under high-phosphate conditions.

Several studies have identified the regulatory role of microRNAs in arterial calcification by directing the complex genetic reprogramming of VSMCs and the functional responses of other relevant cell types for arterial calcification. For the issue about microRNAs regulating arterial calcification, we recommend Leopold’s review [43]. However, the role of miR-670-3p in arterial calcification has not been elucidated yet. By searching the published literature, miR-670-3p could function as a sponge for PP7080 to expedite the proliferation and migration of lung adenocarcinoma cells [27]. Bioinformatics analysis inferred that a low rate of miR-670/KCNS1 ratio in patients with endometrial carcinoma had significantly poorer survival [46]. miR-670-3p also played a crucial role in the development of parthenogenetic activation by reducing the cleavage and blastula rates of embryos [47]. On the other hand, circABCB10 promoted HCC progression by downregulating miR-670-3p and upregulating HMG20A [48]. Briefly, miR-670-3p widely participated in the regulation of tumor development, probably by influencing cell proliferation. Surprisingly, we innovatively discovered the role of EC-derived miR-670-3p in promoting arterial medial calcification both in vivo and in vitro, and revealed the cooperation of two important functional cells in the vascular wall microenvironment to regulate arterial calcification.

IGF-1 is synthesized by almost all tissues and is an important mediator of cell growth, differentiation, and transformation [49]. IGF-1 has a fundamental role in both prenatal and postnatal development and exerts its effects by binding to the IGF-1 receptor [50]. Circulating IGF-1 is generated by the liver under the control of growth hormone. IGF-1 was thought to promote VSMCs proliferation, migration and inhibited apoptosis, thereby increasing plaque stabilization [51]. Accumulated evidence has confirmed the protective effect of IGF-1 against calcification. Shai et al. found that low circulating IGF-I was correlated with atherosclerosis in ApoE-deficient mice [52]. Moreover, serum IGF-1 levels were negatively associated with intima-media thickness and the number of carotid plaques in both the carotid and coronary territories [53]. Radcliff et al. [29] reported that IGF-1 inhibited the spontaneous, as well as bacterial lipopolysaccharide-, TNF-α-, or H_2_O_2_-induced, osteoblastic conversion of calcifying vascular cells. Recent studies indicate that IGF-1 exerted both pleiotropic antioxidant effects and anti-inflammatory effects, which reduced atherosclerotic burden, while losing IGF1R on smooth muscle promoted atherosclerosis [54, 55]. In our research, the luciferase reporter assays confirmed that IGF-1 was the target gene of miR-670-3p. Moreover, downregulation of IGF-1 eliminated the protection effect, thereby aggravating arterial calcification. Consistent with our previous findings, the deposition of the mineralization inhibitors fetuin-A and matrix Gla-protein was also detected to be increased in dialysis vessels [56]. However, there are many remaining questions about the miR-670-3p/IGF-1 axis. Additional studies are required to determine the crosstalk between the IGF-1 system and other growth factors at the level of the ligand-receptor and at the level of post-receptor signaling pathways in vascular diseases.

In conclusion, our present study demonstrated that the ECs original exosomes aggravated high phosphorus-induced arterial calcification in a paracrine manner, which contributed to understanding the pathological mechanism of arterial calcification in patients with uremia. Furthermore, revealing the intricate regulatory network of exosomal miR-670-3p and IGF-1 provided a foundation for developing new diagnostic biomarkers and clinical therapies for arterial calcification in uremic patients.

## Supplementary information

figure1C-runx2

figure1C-β-actin

figure1F-Runx2

figure1F-β-actin

figure2C-CD9

figure2C-CD81

figure2C-tsg101

figure2G-runx2

figure2G-β-actin

figure2I-Runx2

figure2I-β-actin

figure3I-runx2

figure3I-β-actin

figure4D-igf1

figure4D-β-actin

figure4E-IGF1

figure4E-β-actin

figure4G-Runx2

figure4G-GAPDH

figure4H-IGF1-1

figure4H-β-actin

figure4J-Runx2

figure4J-β-actin

supplemental files

reproducibility checklist

## Data Availability

All data are included in this article and its supplementary materials or available upon request to the corresponding author.
