# Supplementary figures and images for: The crosstalk between endothelial cells and vascular smooth muscle cells aggravates high phosphorus-induced arterial calcification
Source: Cell Death Dis. 2022 Jul 26;13(7):650. doi: 10.1038/s41419-022-05064-5 (PMC9325771; doi:10.1038/s41419-022-05064-5)

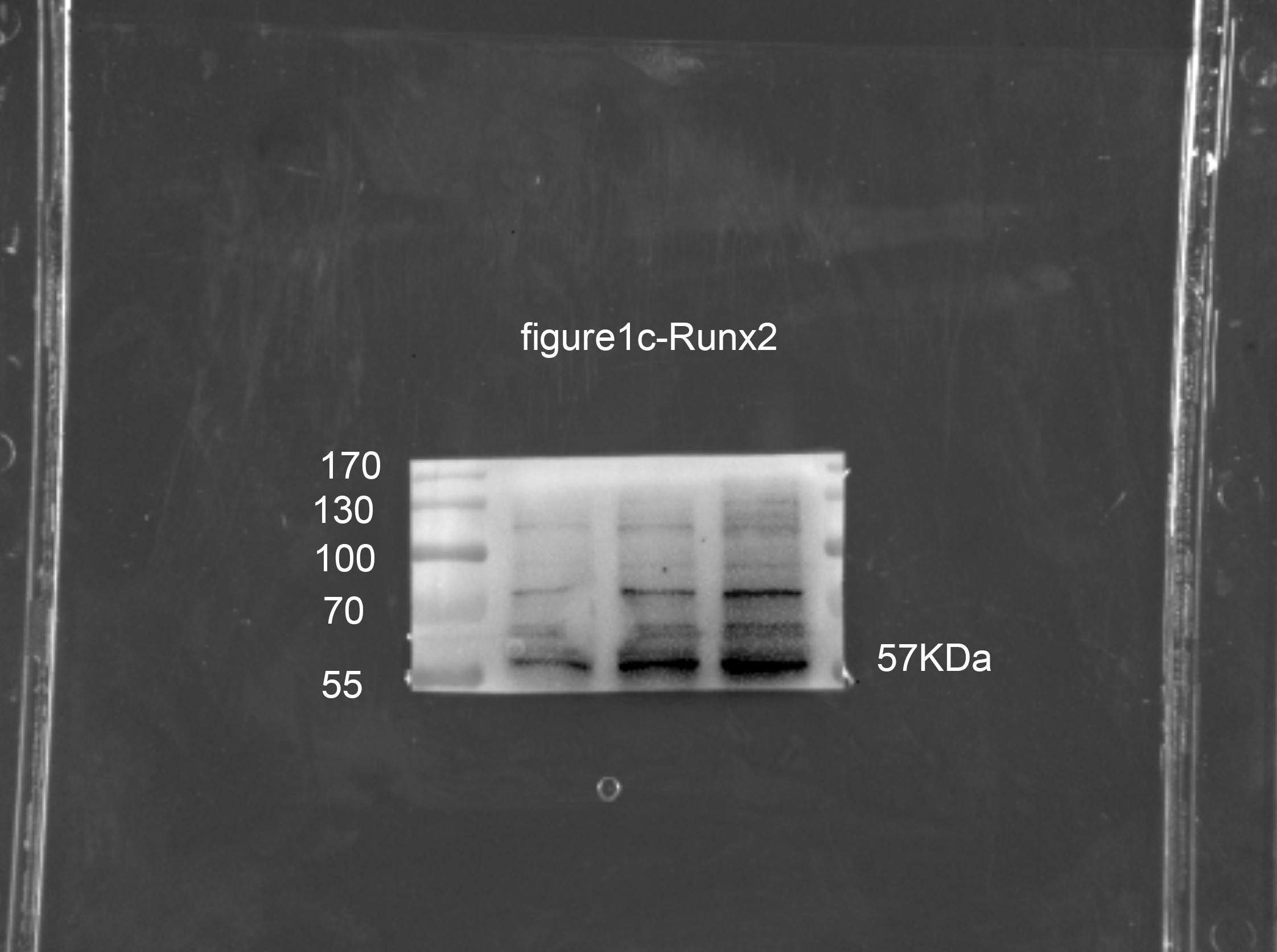

Supplement: Supplementary file 1 — figure1C-runx2 [file 41419_2022_5064_MOESM1_ESM.tif]

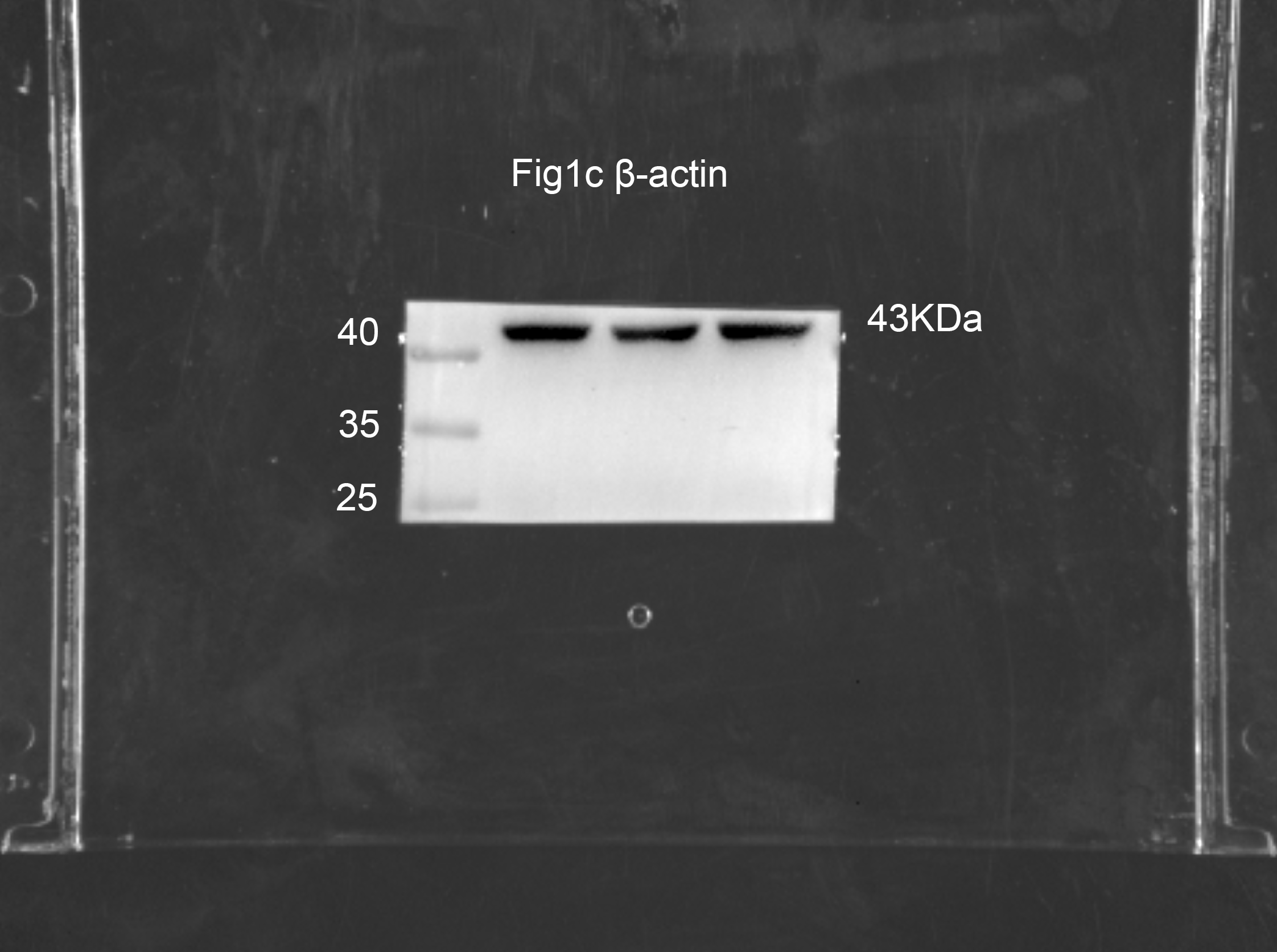

Supplement: Supplementary file 2 — figure1C-β-actin [file 41419_2022_5064_MOESM2_ESM.tif]

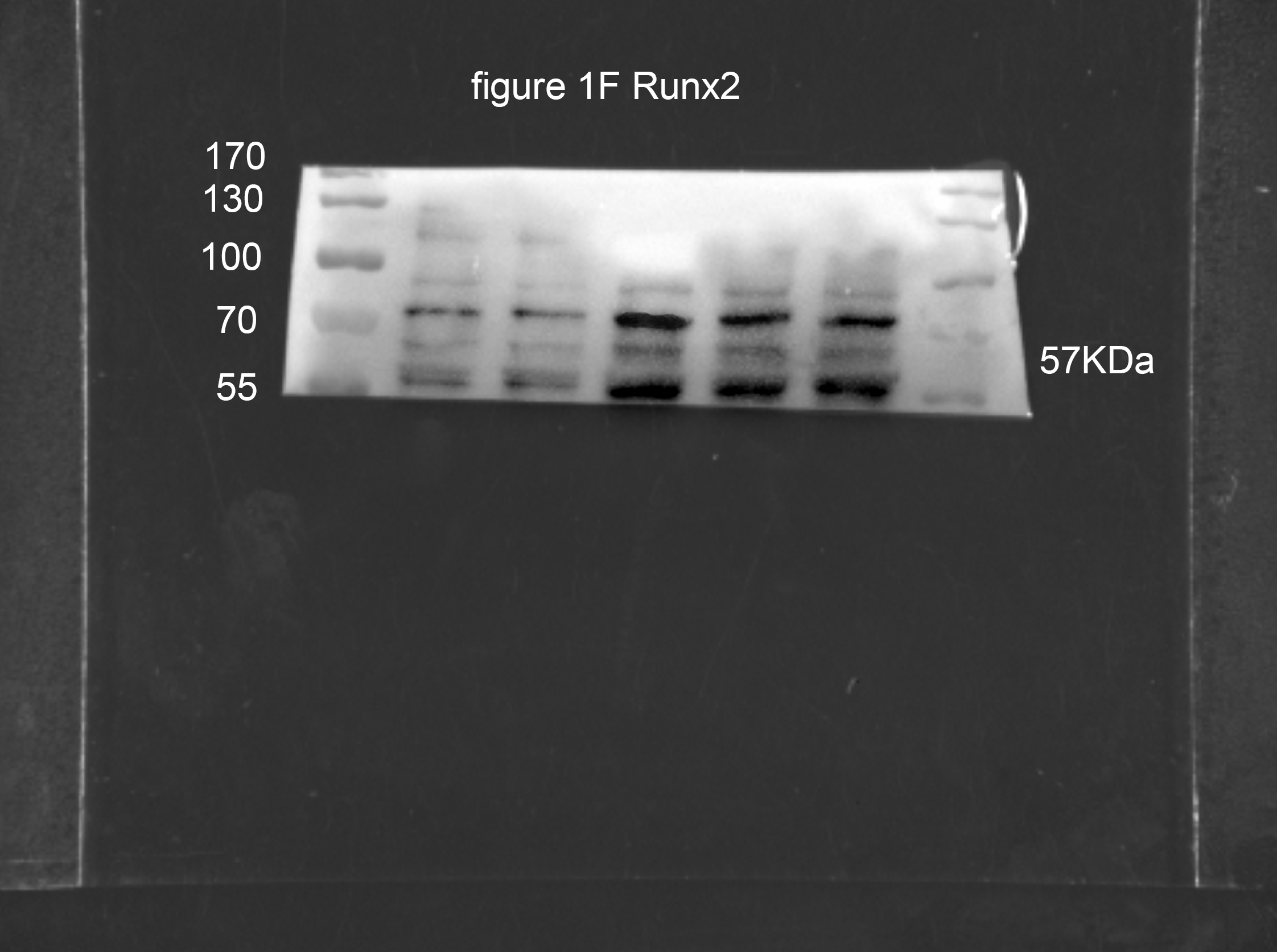

Supplement: Supplementary file 3 — figure1F-Runx2 [file 41419_2022_5064_MOESM3_ESM.tif]

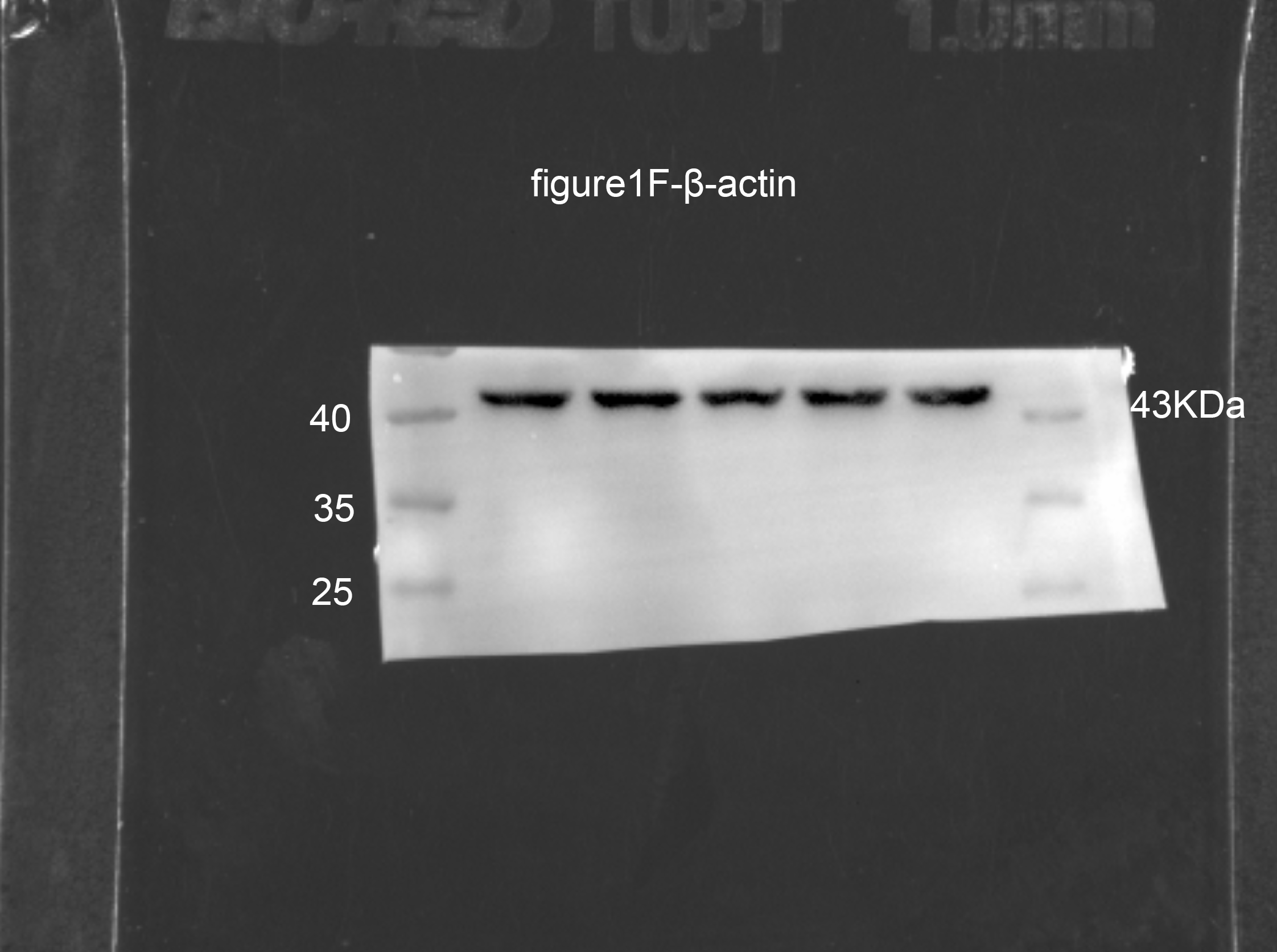

Supplement: Supplementary file 4 — figure1F-β-actin [file 41419_2022_5064_MOESM4_ESM.tif]

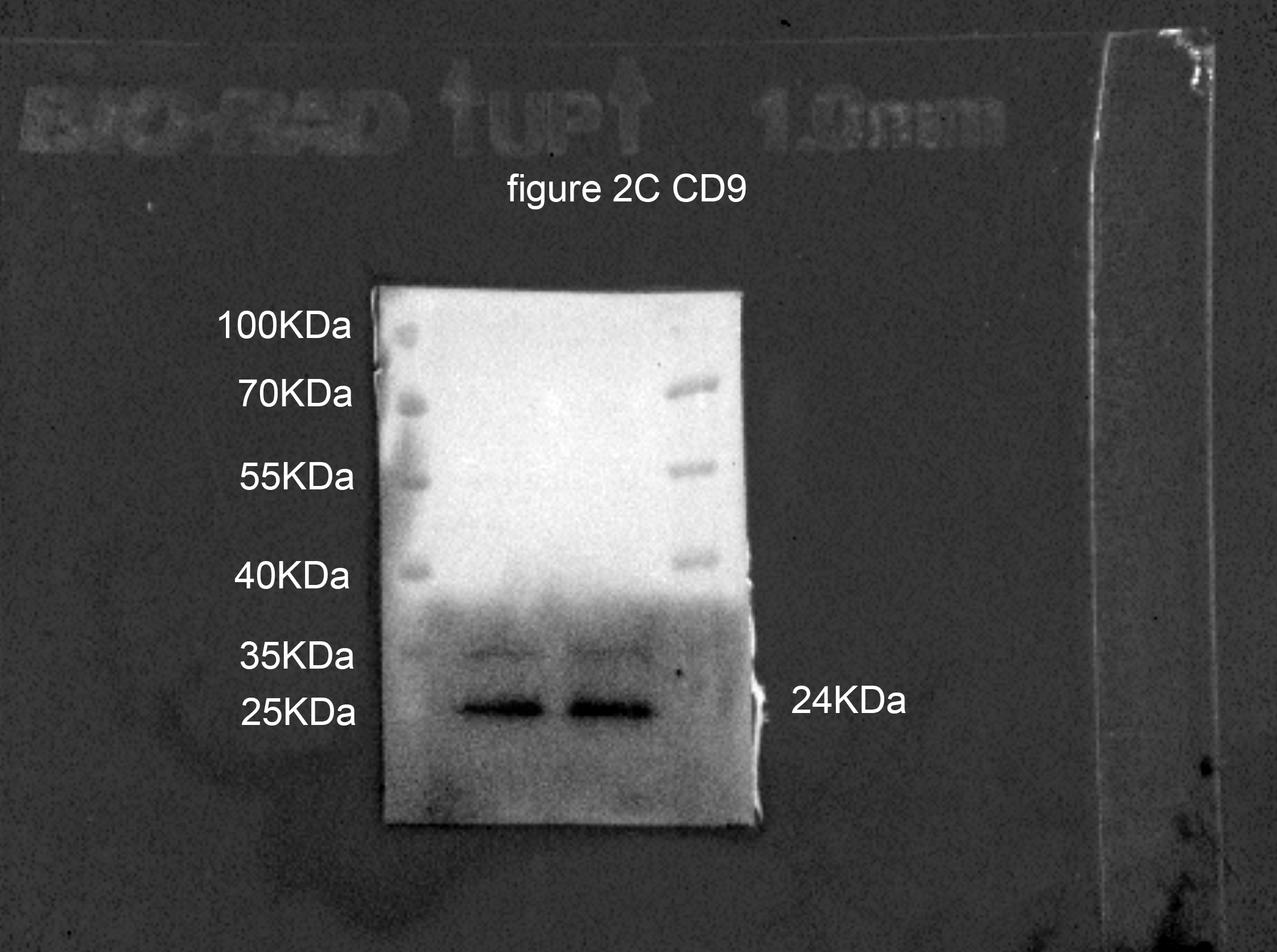

Supplement: Supplementary file 5 — figure2C-CD9 [file 41419_2022_5064_MOESM5_ESM.tif]

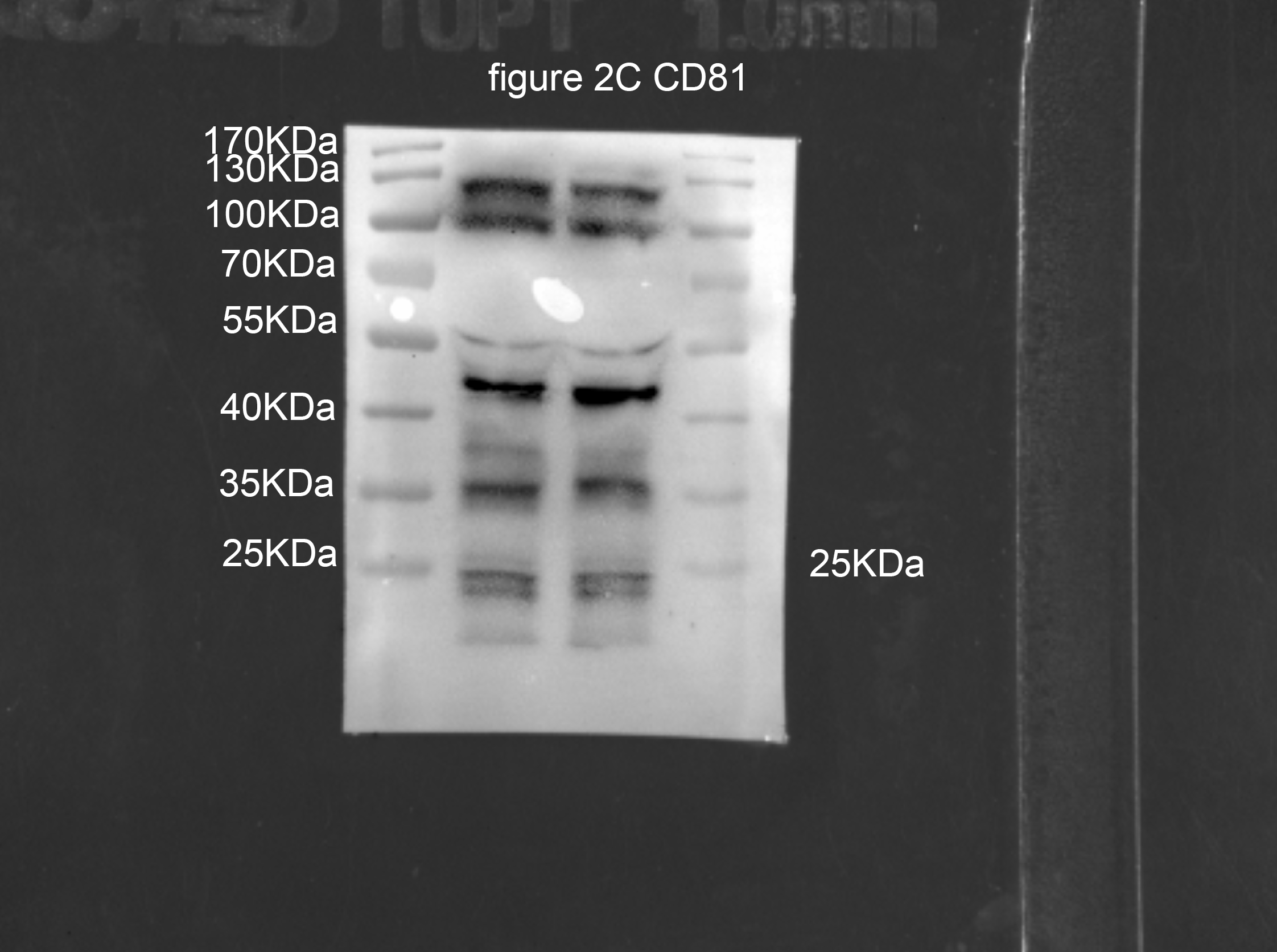

Supplement: Supplementary file 6 — figure2C-CD81 [file 41419_2022_5064_MOESM6_ESM.tif]

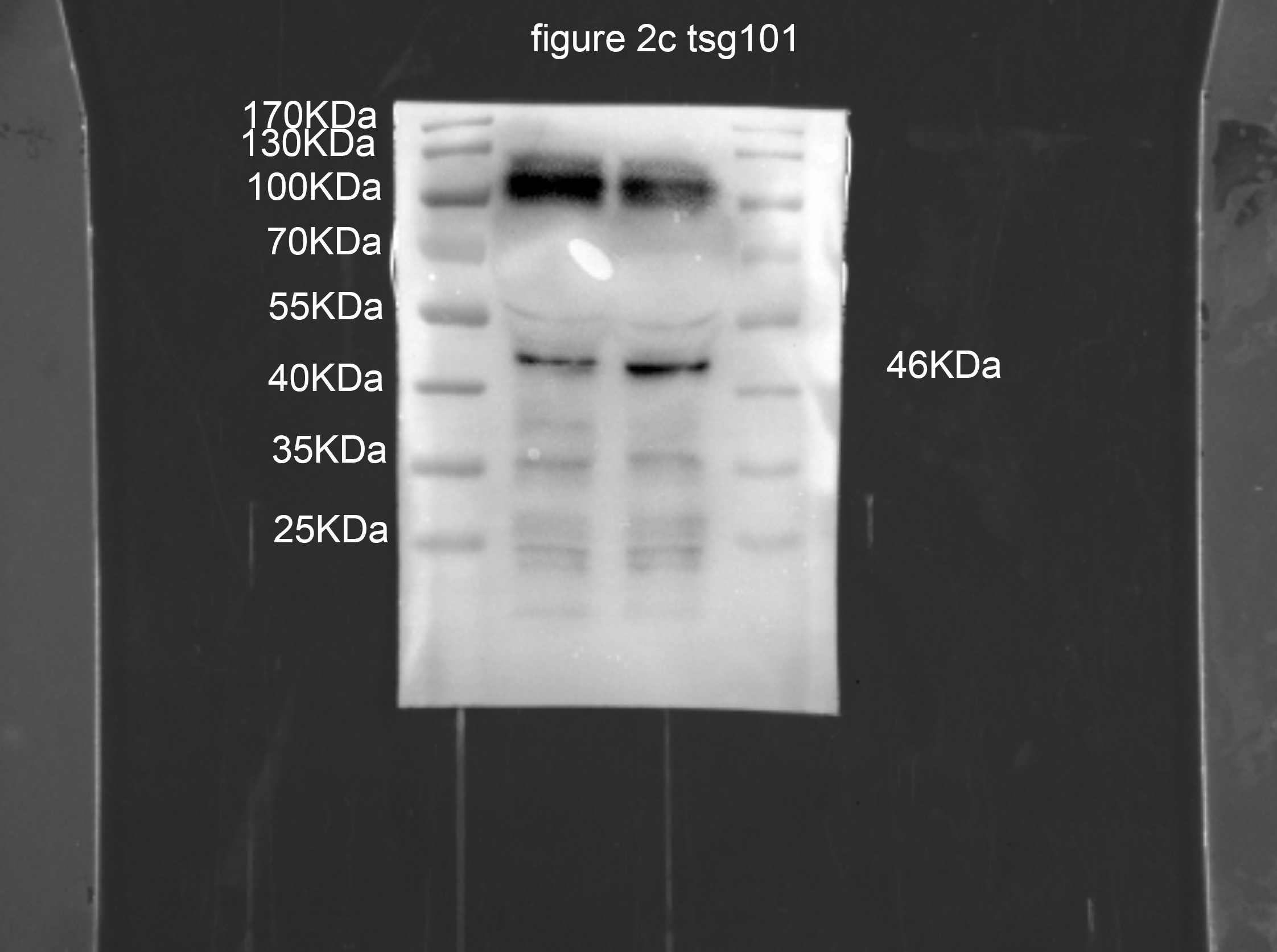

Supplement: Supplementary file 7 — figure2C-tsg101 [file 41419_2022_5064_MOESM7_ESM.tif]

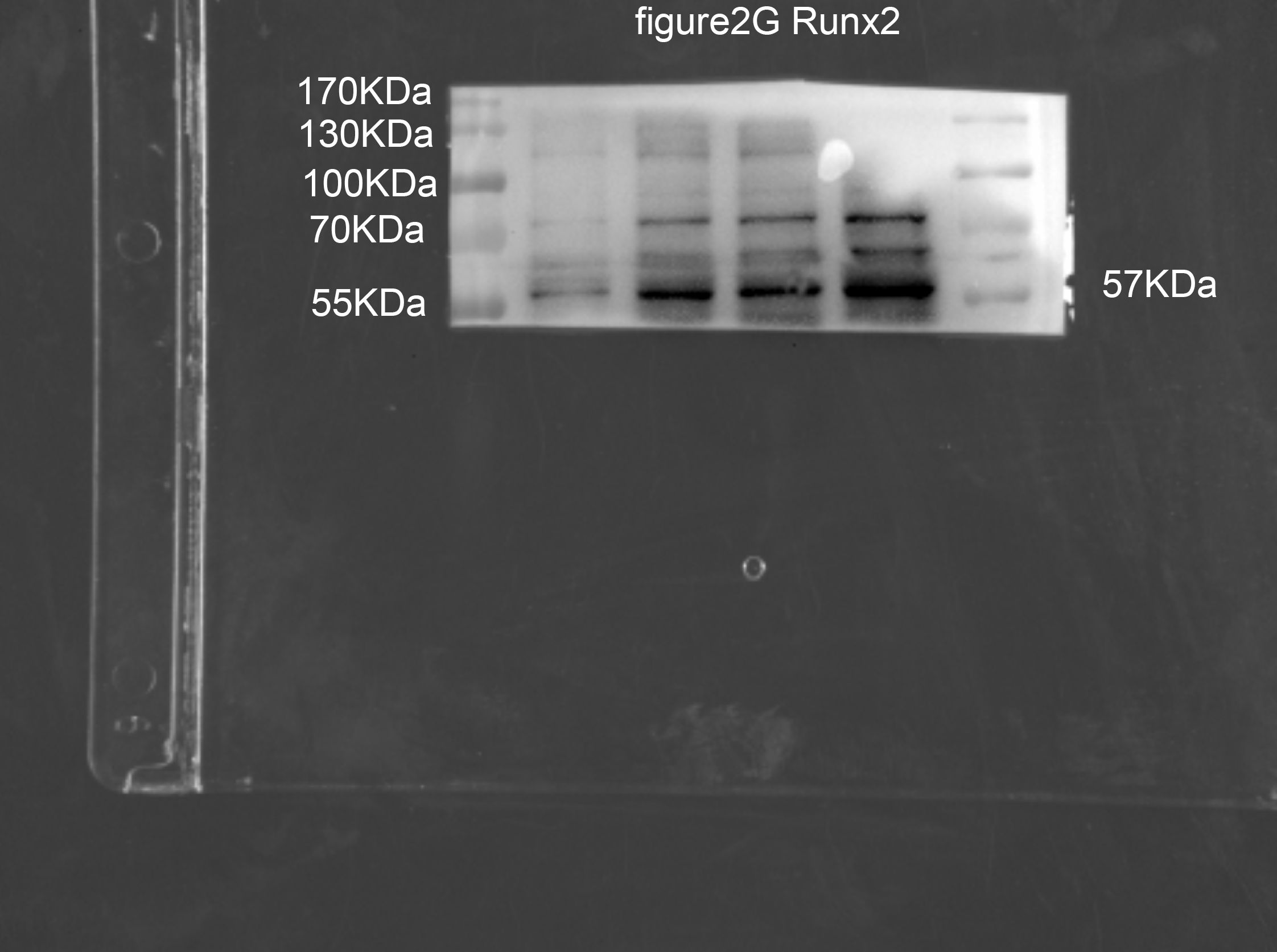

Supplement: Supplementary file 8 — figure2G-runx2 [file 41419_2022_5064_MOESM8_ESM.tif]

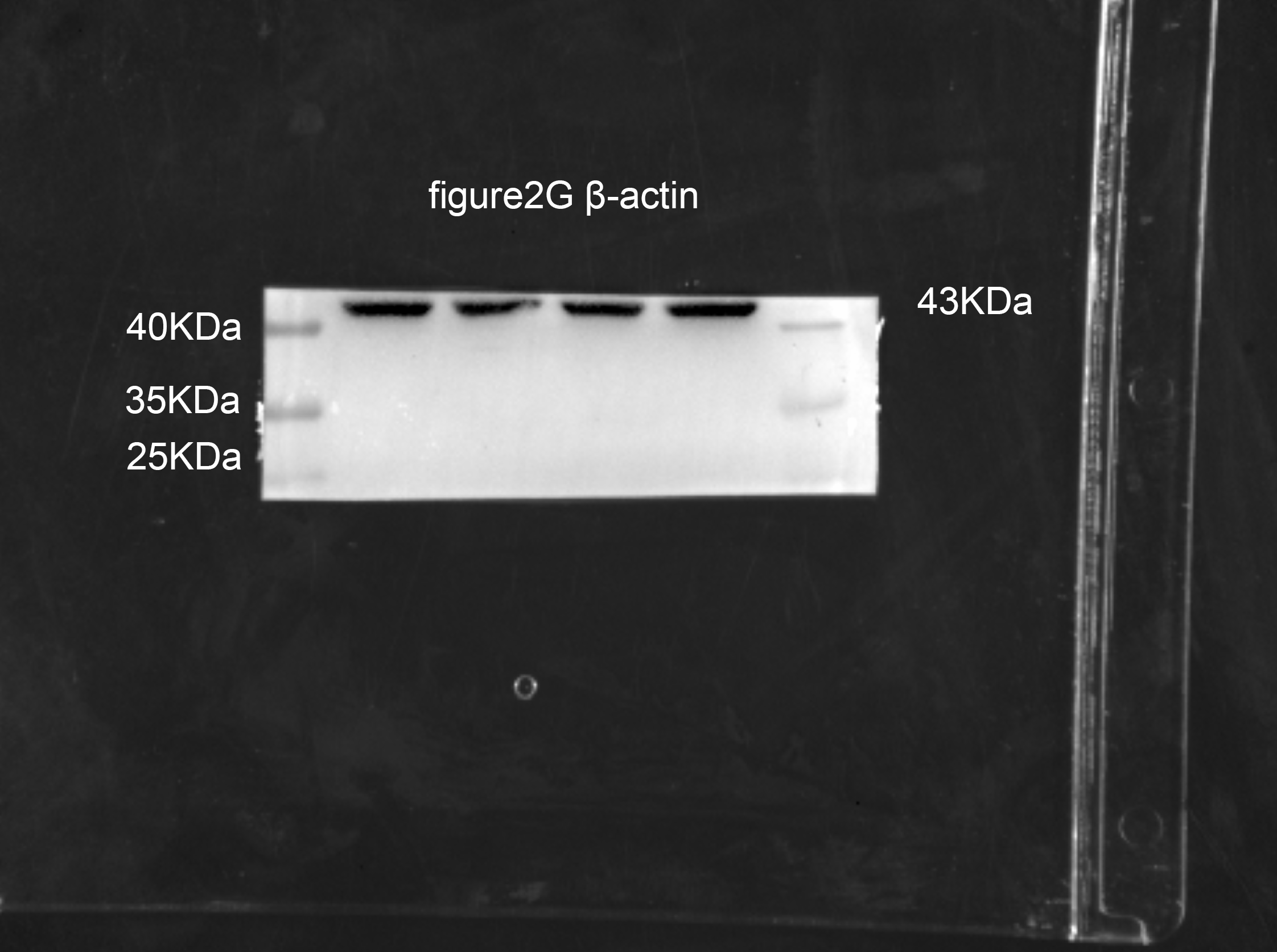

Supplement: Supplementary file 9 — figure2G-β-actin [file 41419_2022_5064_MOESM9_ESM.tif]

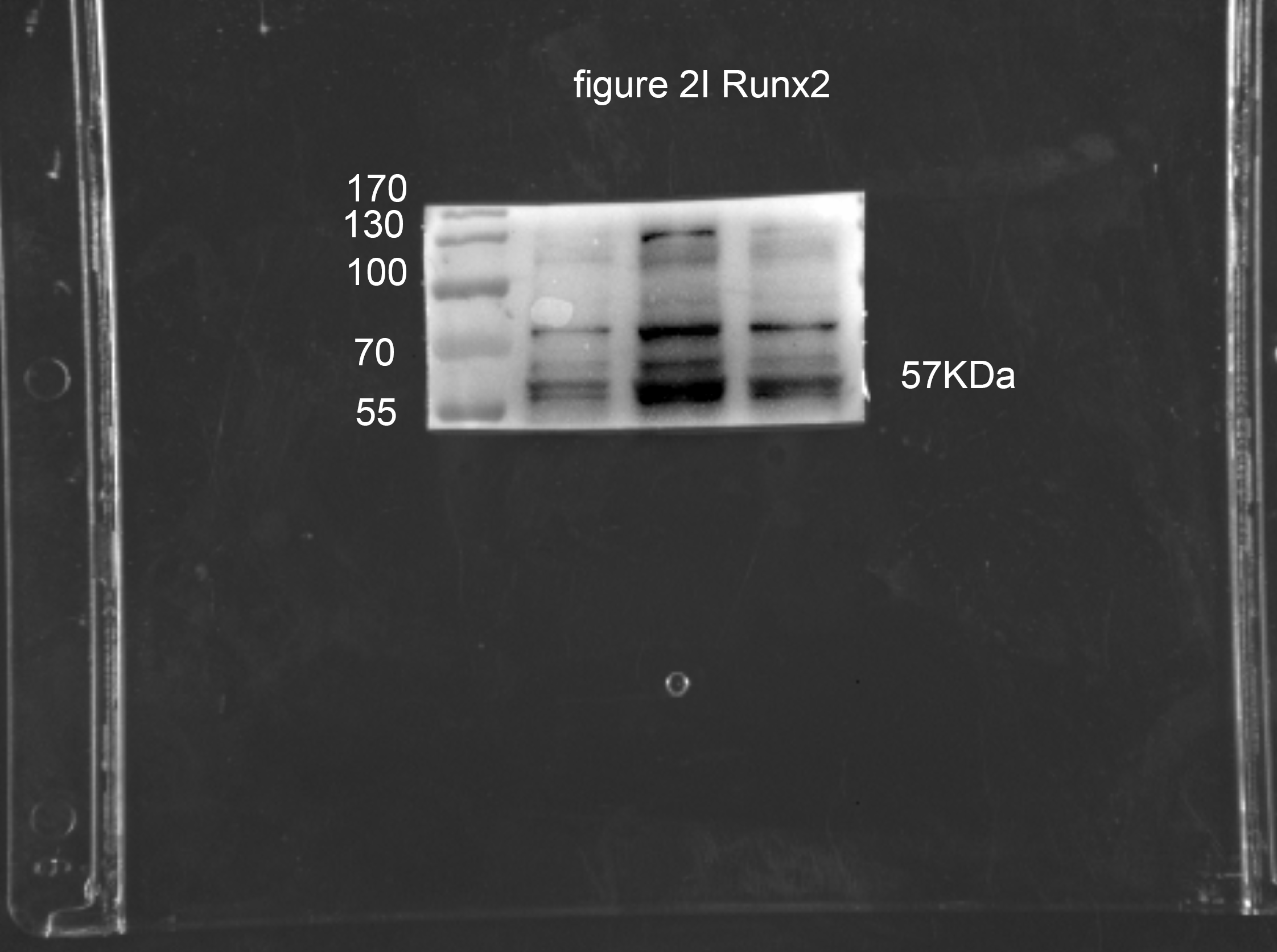

Supplement: Supplementary file 10 — figure2I-Runx2 [file 41419_2022_5064_MOESM10_ESM.tif]

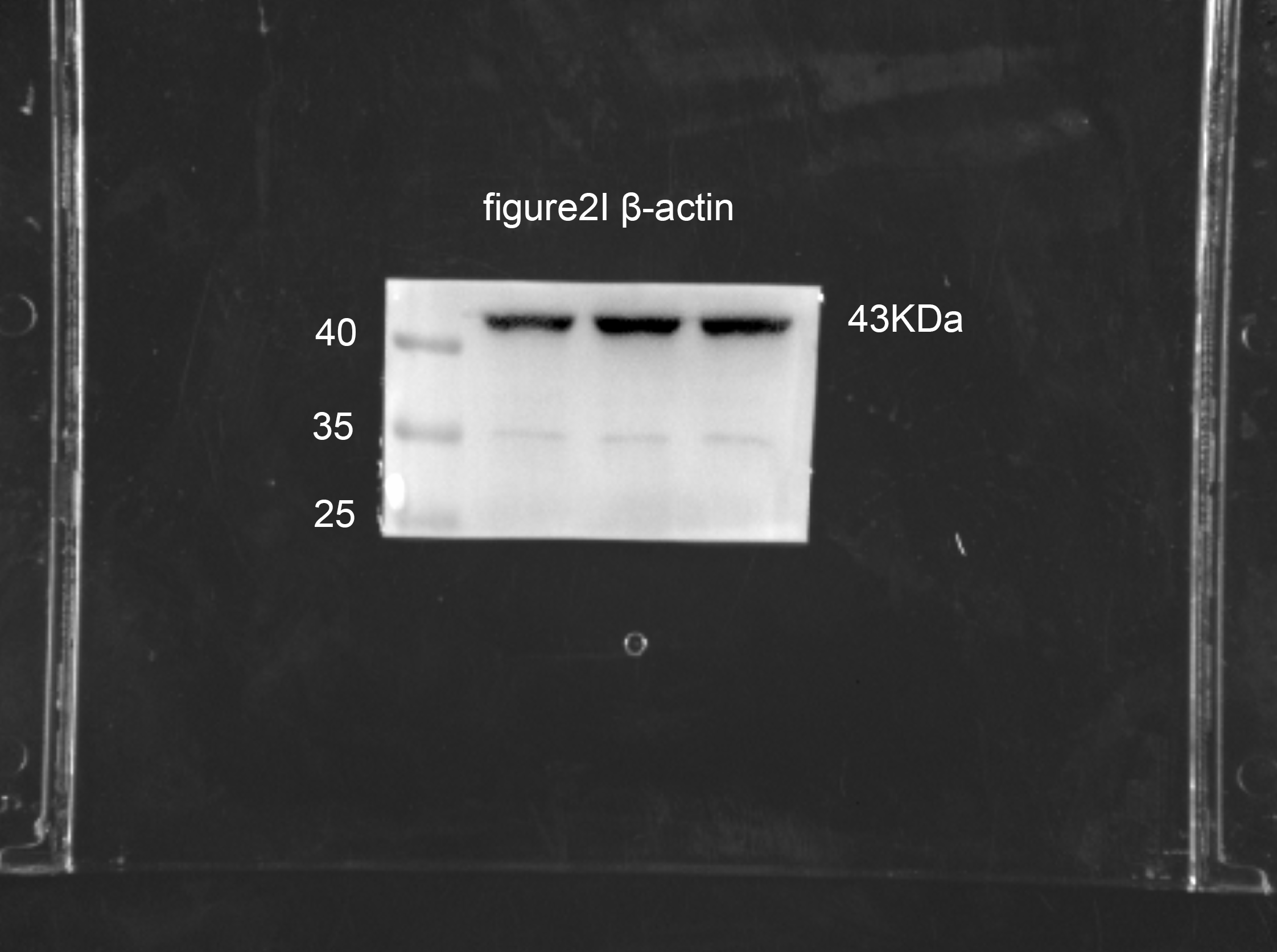

Supplement: Supplementary file 11 — figure2I-β-actin [file 41419_2022_5064_MOESM11_ESM.tif]

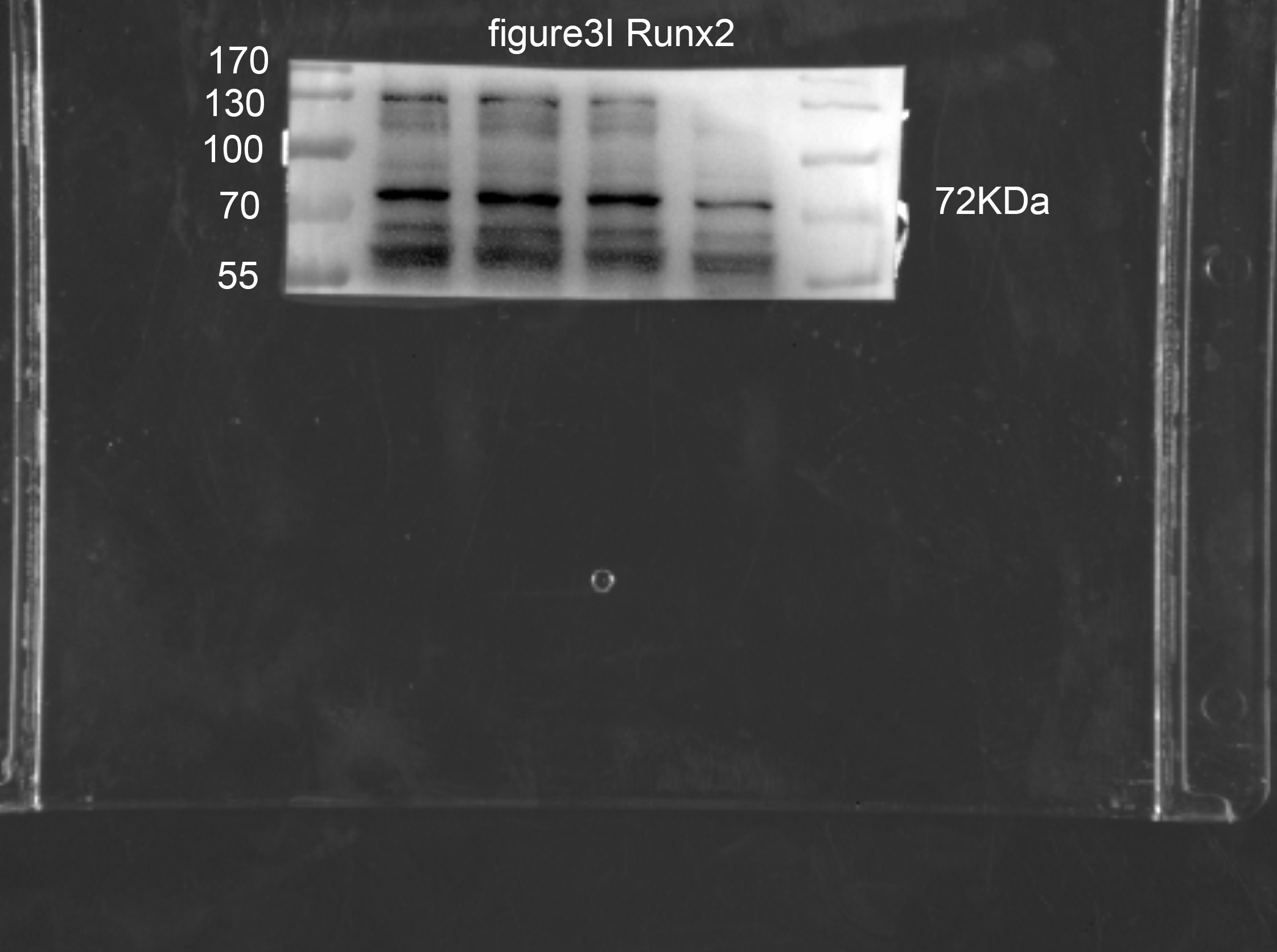

Supplement: Supplementary file 12 — figure3I-runx2 [file 41419_2022_5064_MOESM12_ESM.tif]

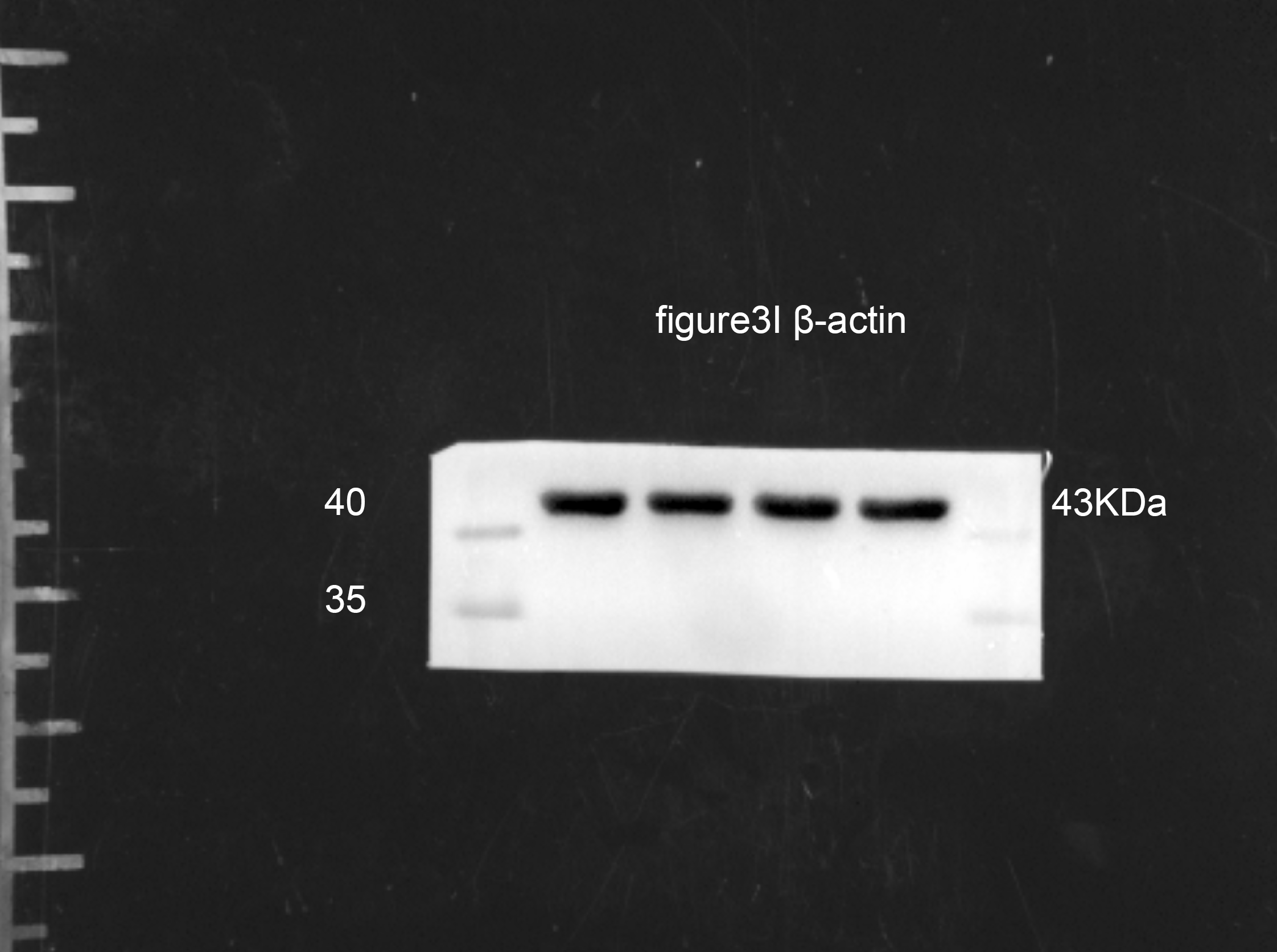

Supplement: Supplementary file 13 — figure3I-β-actin [file 41419_2022_5064_MOESM13_ESM.tif]

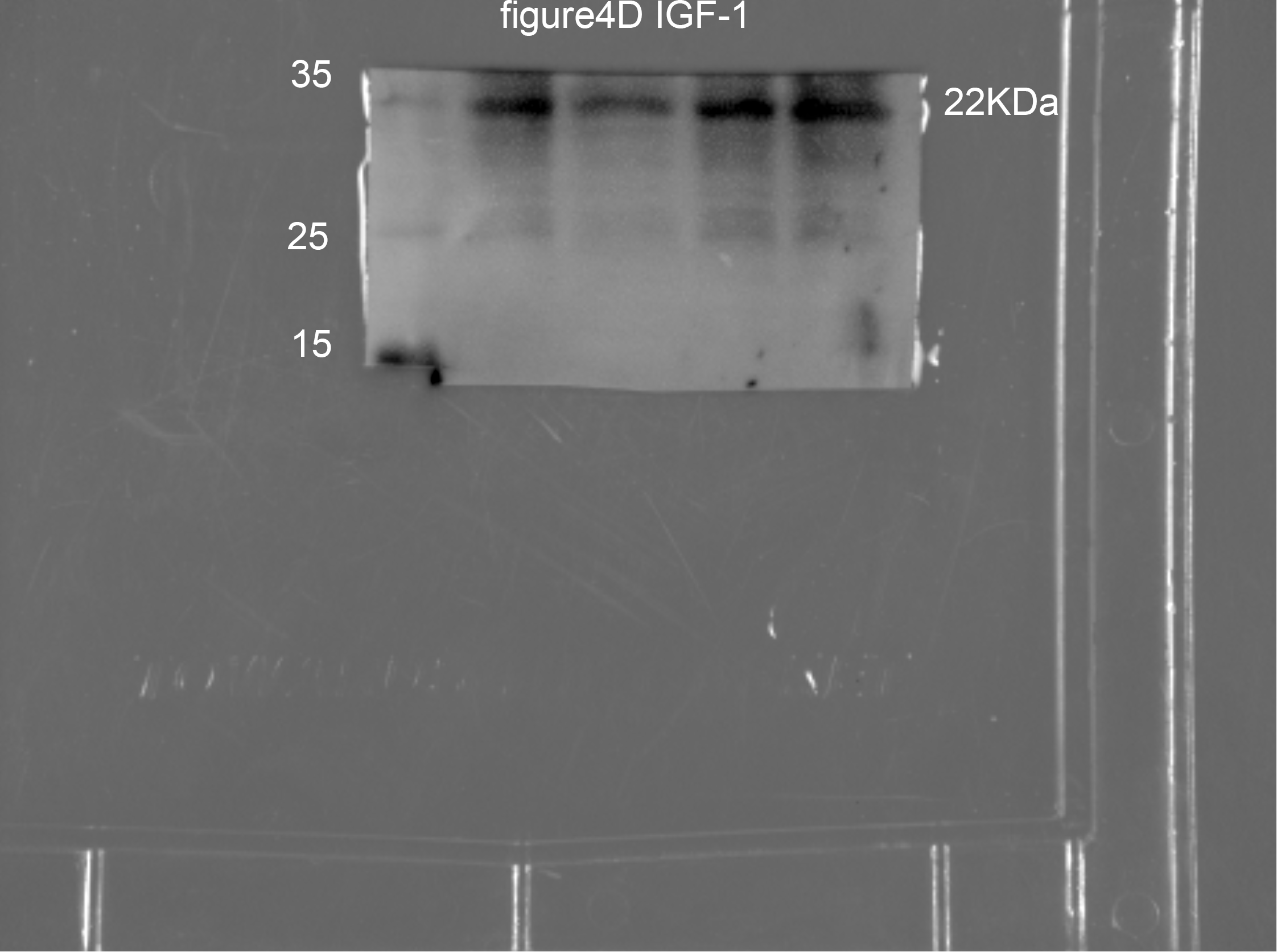

Supplement: Supplementary file 14 — figure4D-igf1 [file 41419_2022_5064_MOESM14_ESM.tif]

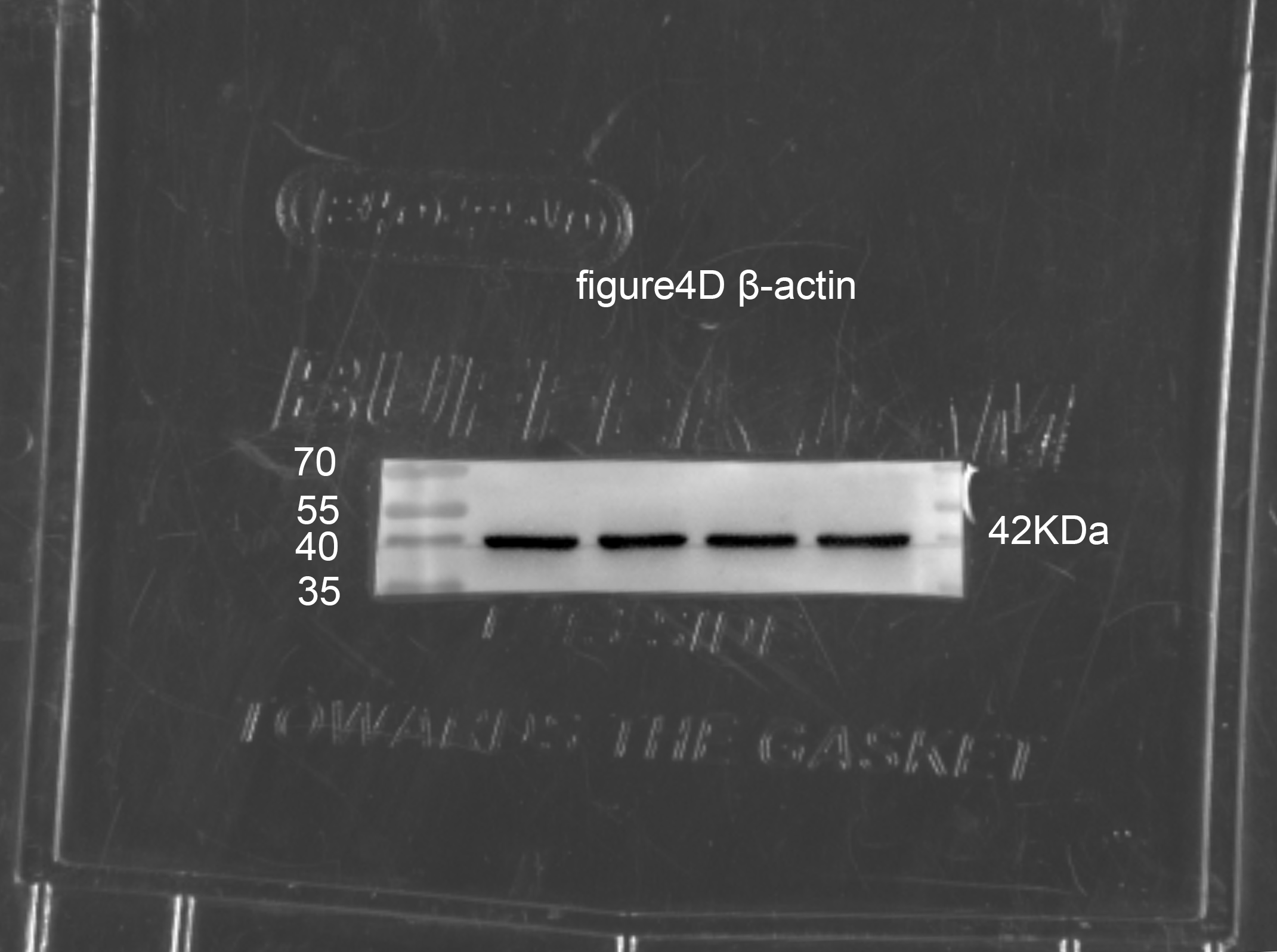

Supplement: Supplementary file 15 — figure4D-β-actin [file 41419_2022_5064_MOESM15_ESM.tif]

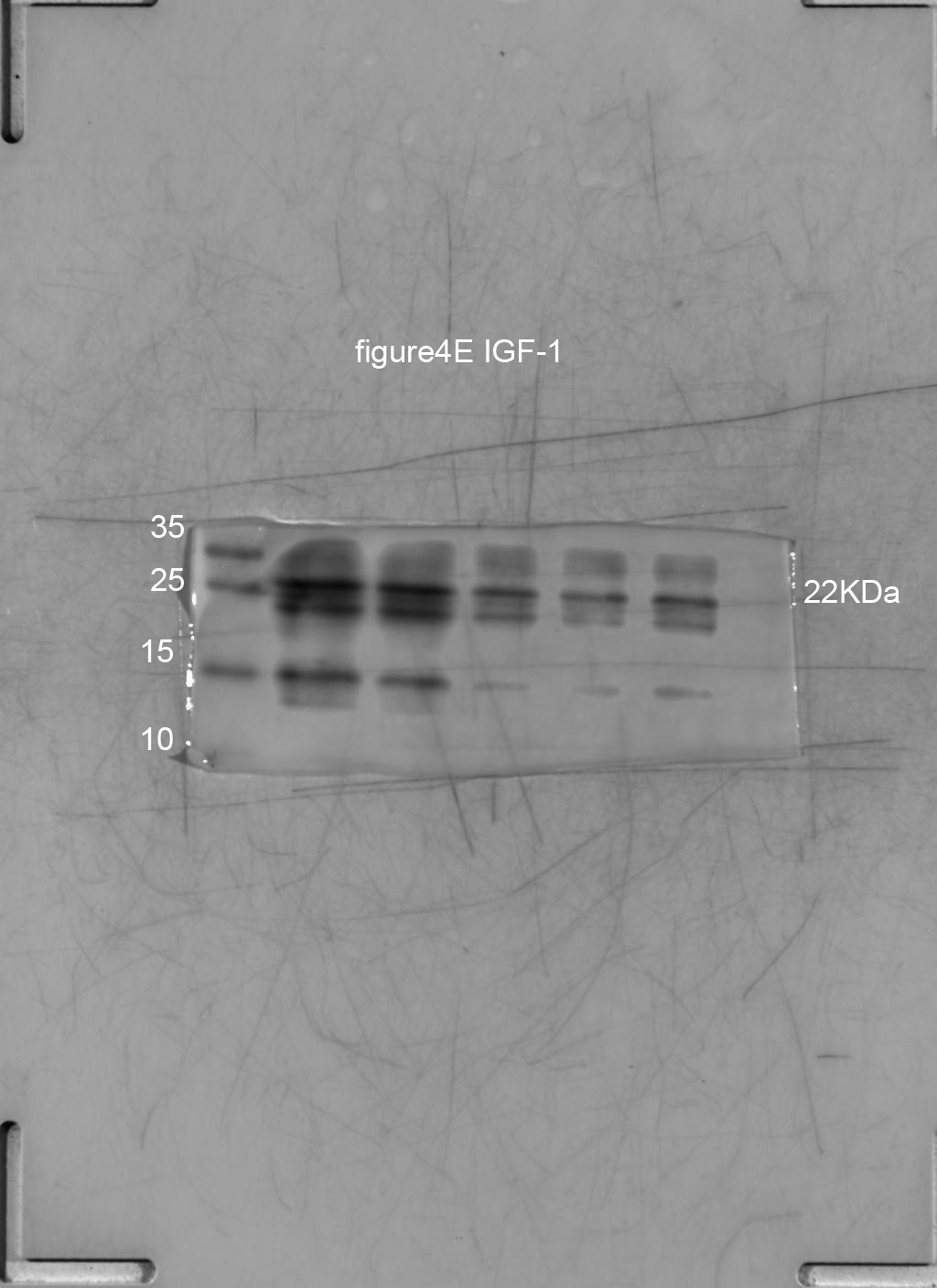

Supplement: Supplementary file 16 — figure4E-IGF1 [file 41419_2022_5064_MOESM16_ESM.tif]

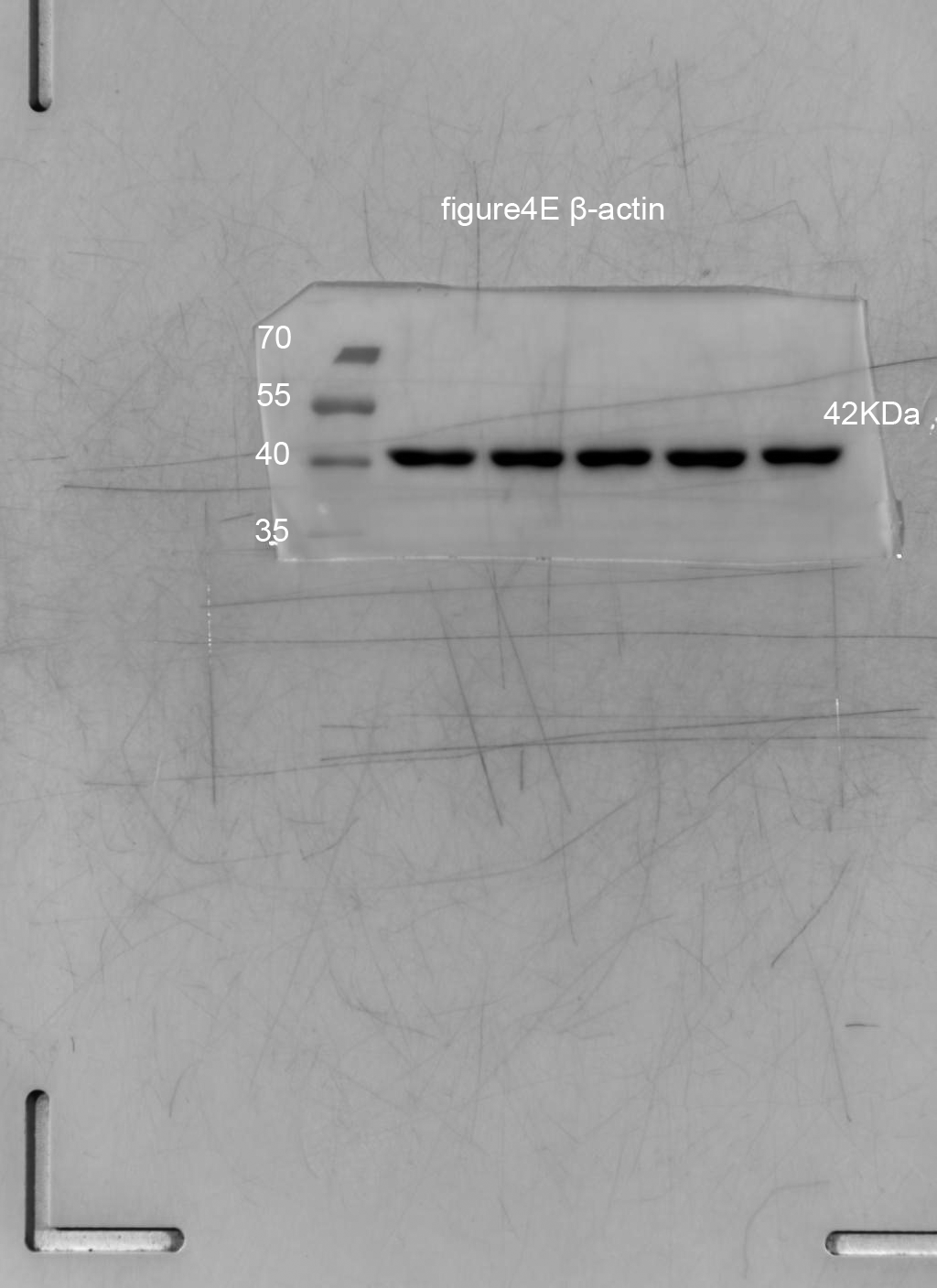

Supplement: Supplementary file 17 — figure4E-β-actin [file 41419_2022_5064_MOESM17_ESM.tif]

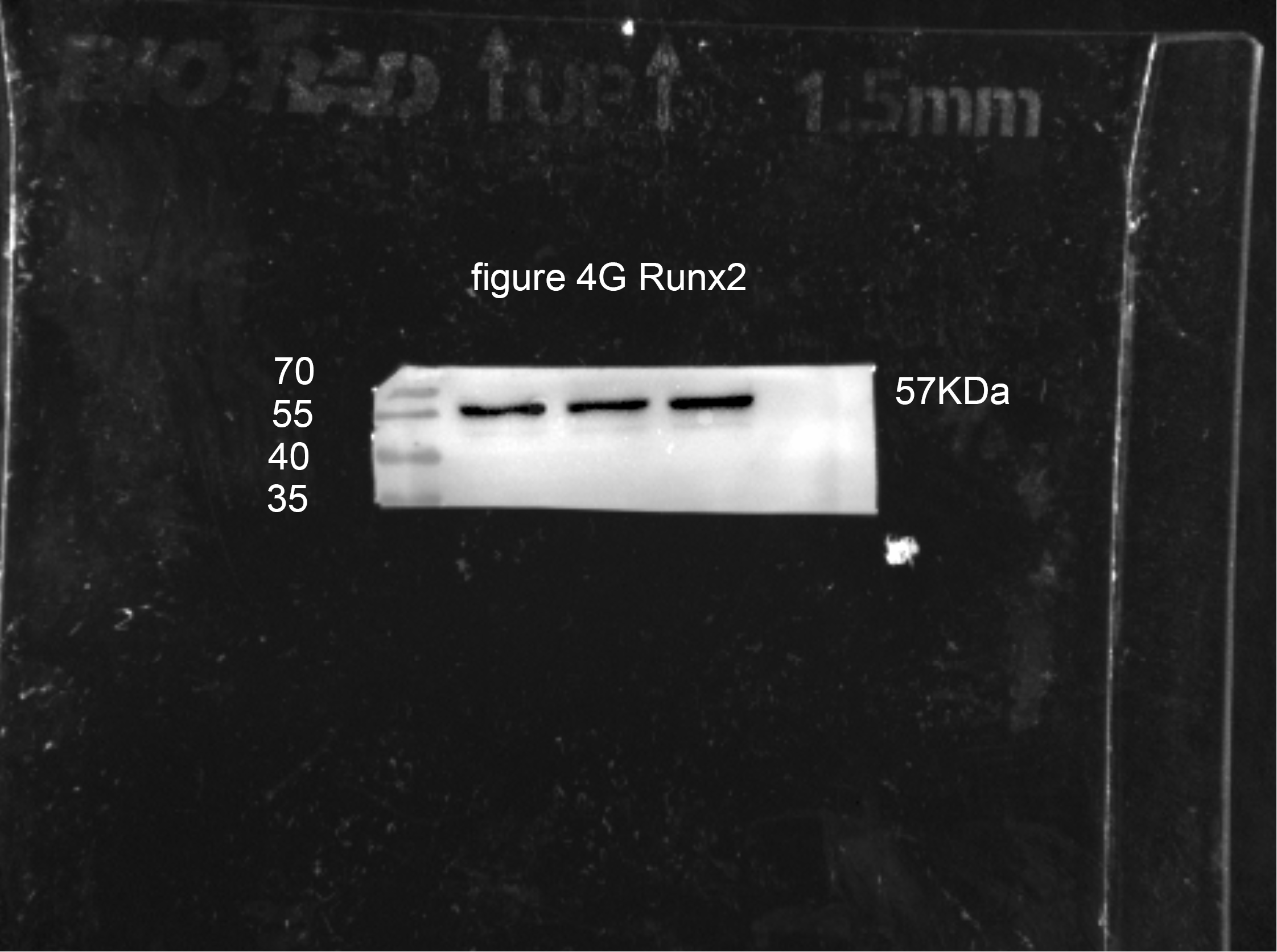

Supplement: Supplementary file 18 — figure4G-Runx2 [file 41419_2022_5064_MOESM18_ESM.tif]

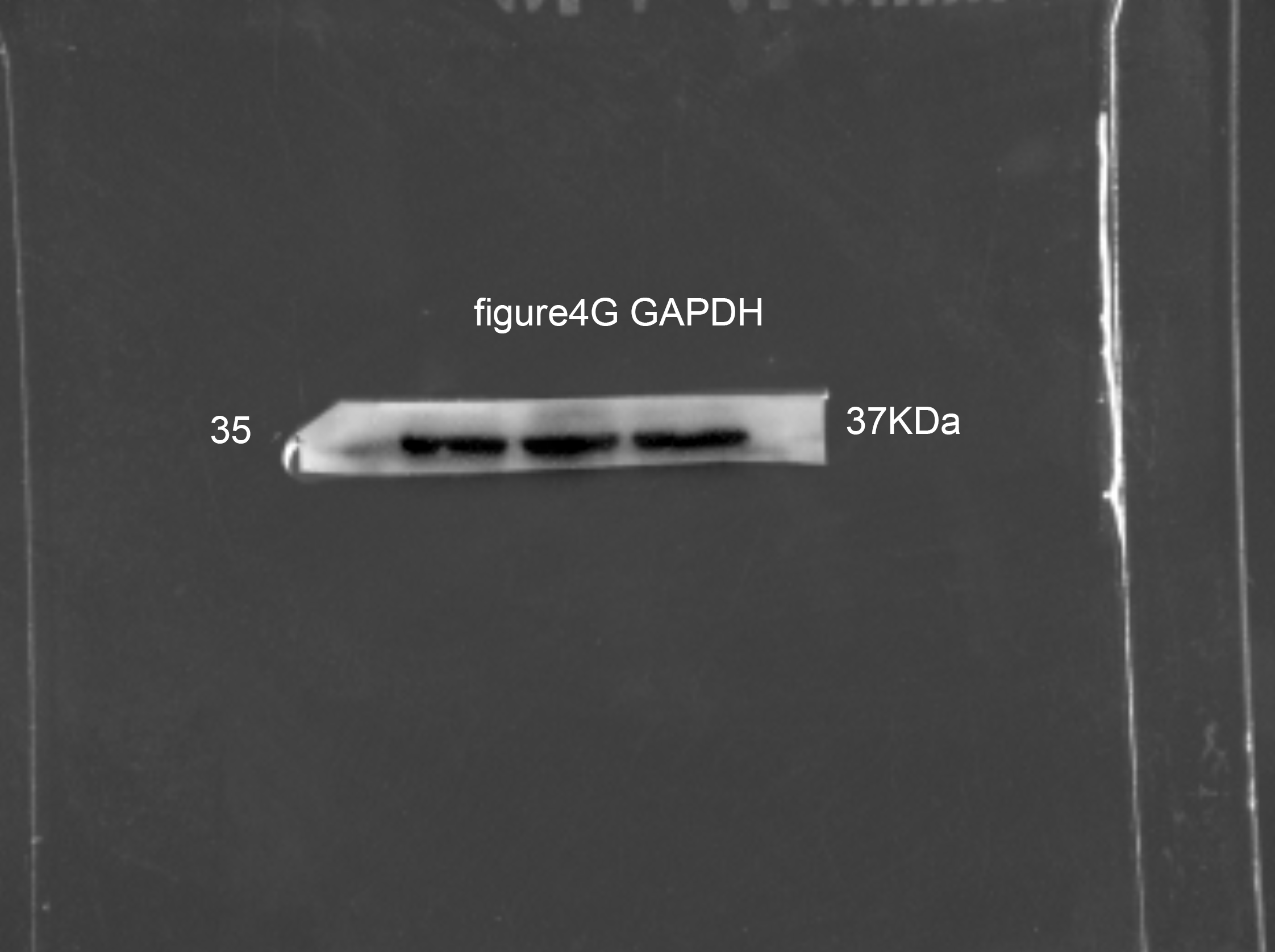

Supplement: Supplementary file 19 — figure4G-GAPDH [file 41419_2022_5064_MOESM19_ESM.tif]

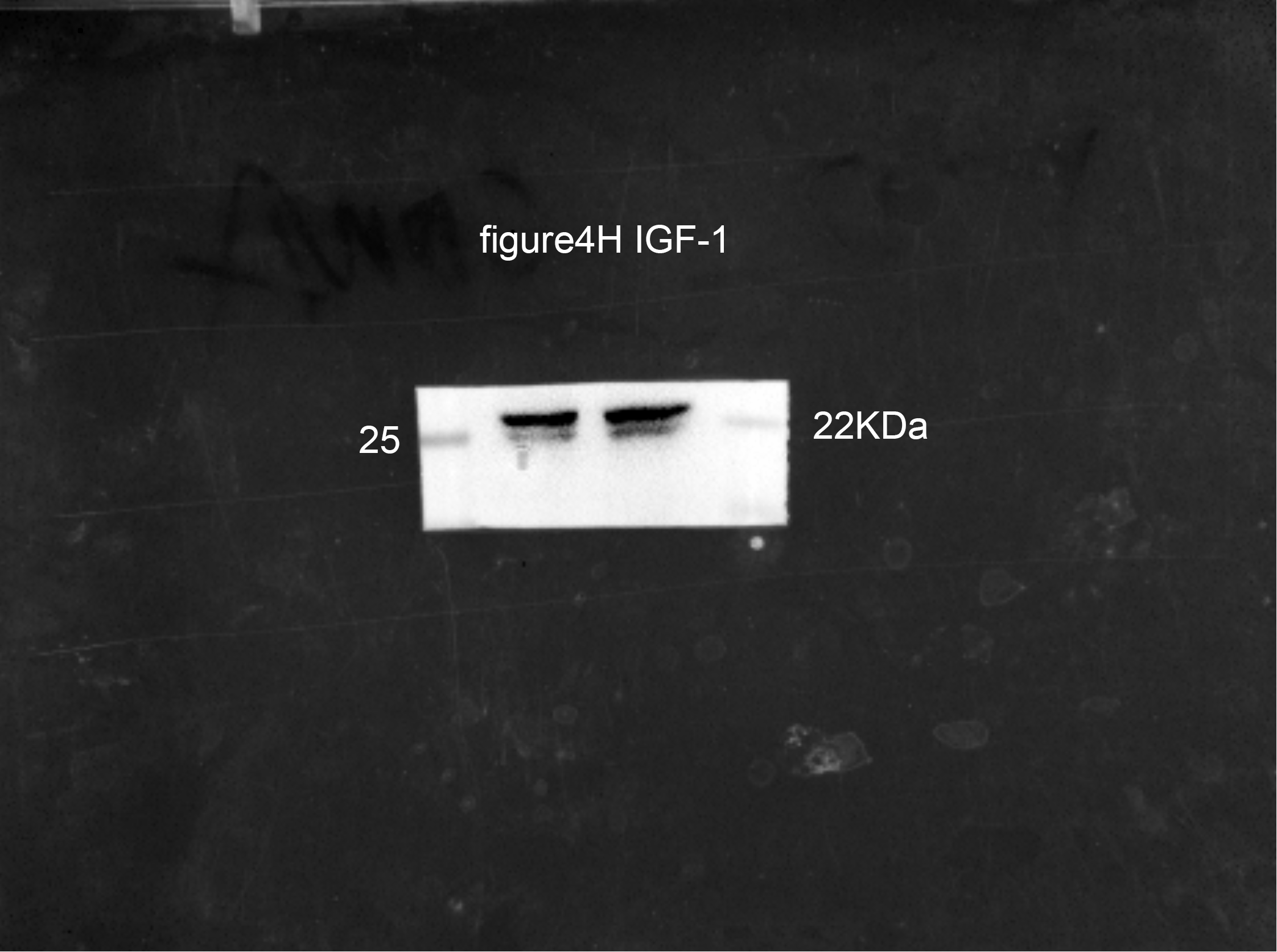

Supplement: Supplementary file 20 — figure4H-IGF1-1 [file 41419_2022_5064_MOESM20_ESM.tif]

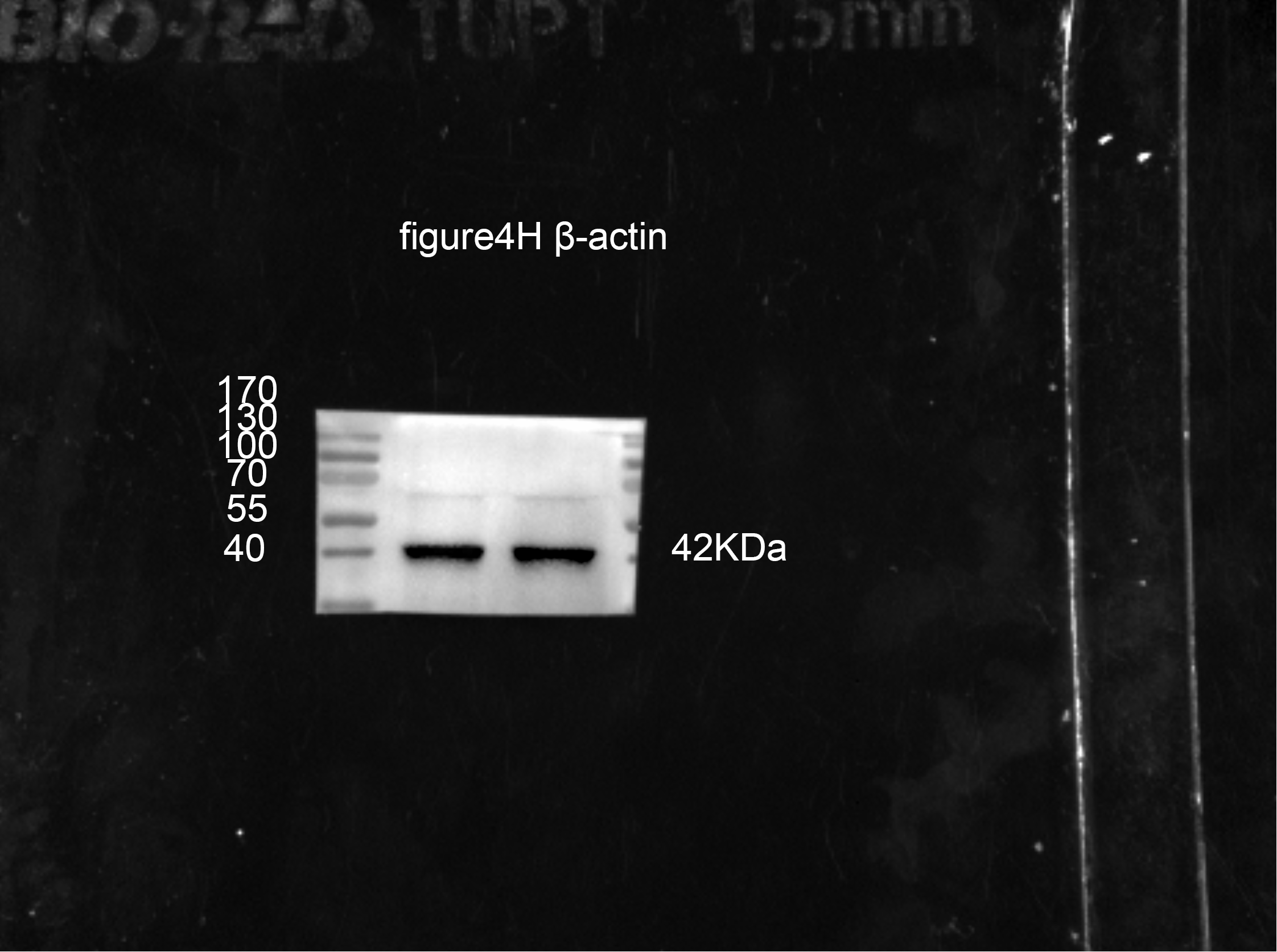

Supplement: Supplementary file 21 — figure4H-β-actin [file 41419_2022_5064_MOESM21_ESM.tif]

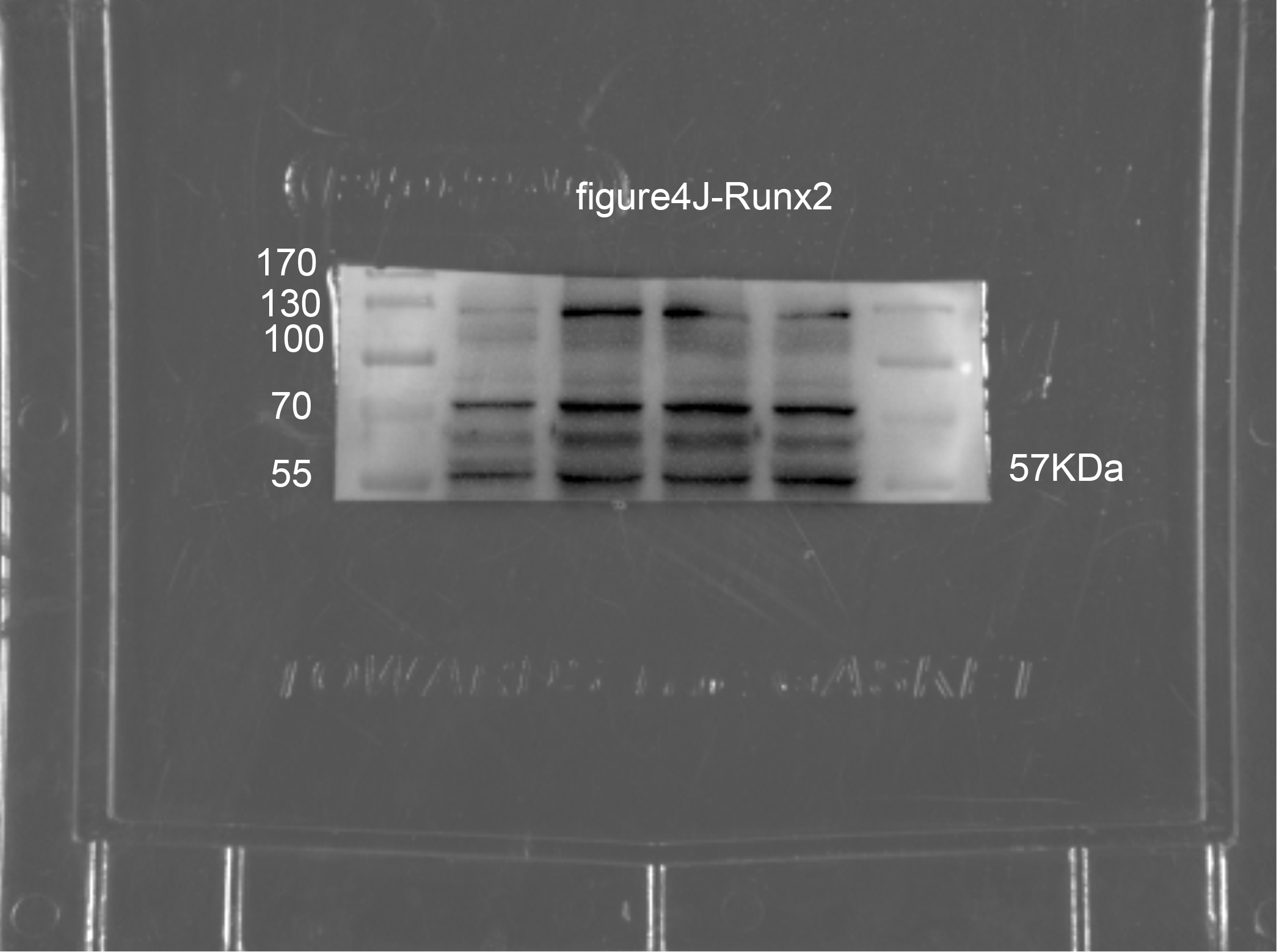

Supplement: Supplementary file 22 — figure4J-Runx2 [file 41419_2022_5064_MOESM22_ESM.tif]

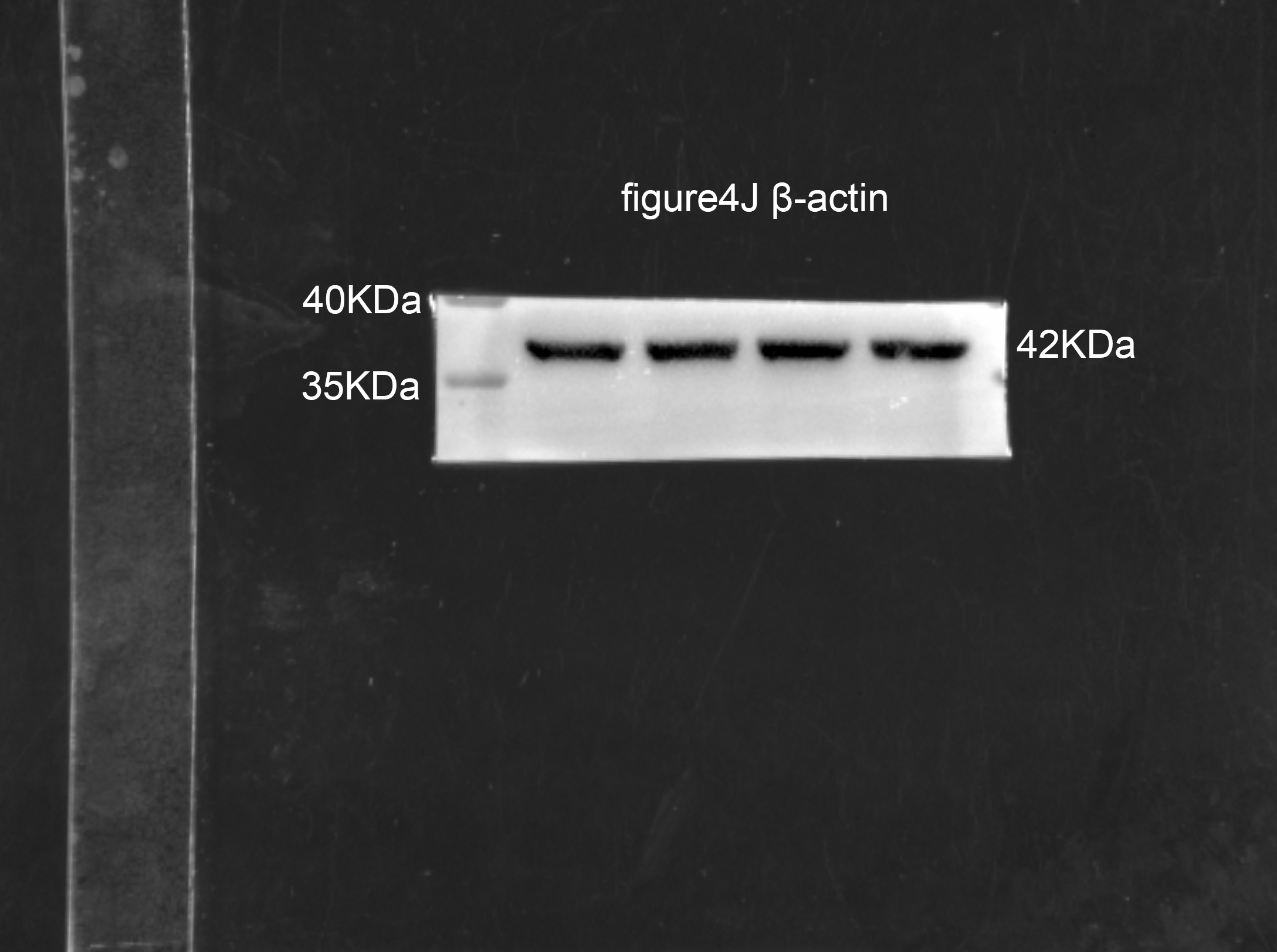

Supplement: Supplementary file 23 — figure4J-β-actin [file 41419_2022_5064_MOESM23_ESM.tif]
