# Supplementary material for: The crosstalk between endothelial cells and vascular smooth muscle cells aggravates high phosphorus-induced arterial calcification
Source: Cell Death Dis. 2022 Jul 26;13(7):650. doi: 10.1038/s41419-022-05064-5 (PMC9325771; doi:10.1038/s41419-022-05064-5)
Supplement: Supplementary file 24 — supplemental files [file 41419_2022_5064_MOESM24_ESM.docx]

Supplemental Data

Supplemental method

**Viability assay**

In brief, cells (2 × 10^3^ cells per well; five replicates per group) were seeded into 96-well culture plates and treated with exosomes (100 μg/mL) from

different groups or PBS. A group without cells served as the blank. After 24h,48h and 72h, 10 μL cell counting kit-8 reagent (CCK-8, Dojindo, Kyushu Island, Japan) was added to the culture medium(100 μL per well), then incubated at 37 °C for 3 h. Cell viability was detected at 450 nm (Bio-Tek 680, Vermont, USA).

Supplemental Figures


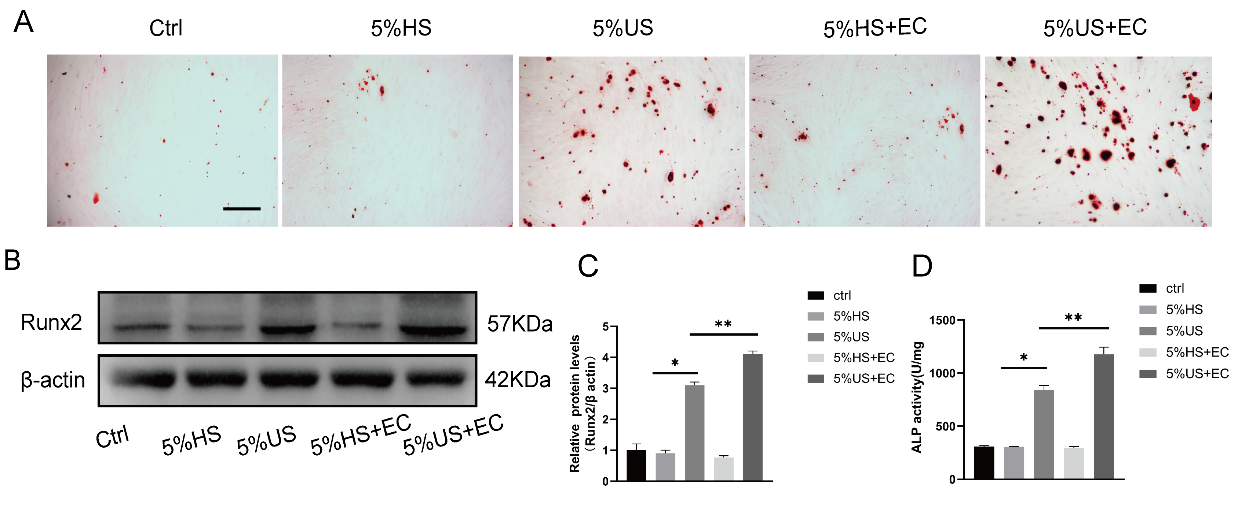


Supplemental Figure 1. The effect of uremia serum on VSMCs calcification. (A-C) 5% uremia serum (US) and 5% healthy serum (HS) were supplemented to the culture media (CM) of VSMCs; the US and HS treated EC CM were also collected for VSMCs cultures. Alizarin Red S staining (A), Runx2 expression (B, C) and ALP activity (D) were measured on day 14, day 7 and day 7, respectively (Bar = 100 μm). Data are shown as mean ± SD with three independent experiments. **p* < 0.05, ***p* < 0.01.

Supplemental Figure 2


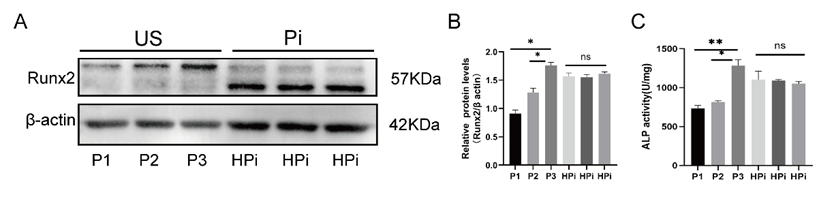


Supplemental Figure 2. Uremia serum and high phosphorus induced VSMCs calcification. (A-C) VSMCs were incubated with US and the same levels of high phosphorus for 48 h, respectively. (A, B) The expression of Runx2 was determined by Western blotting in VSMCs incubated with US and Pi. (C)ALP activity was measured by an ALP kit in VSMCs incubated with US and Pi. Data are shown as mean ± SD, with three independent experiments. ns: no significance, **p* < 0.05, ***p* < 0.01.

Supplemental Figure 3


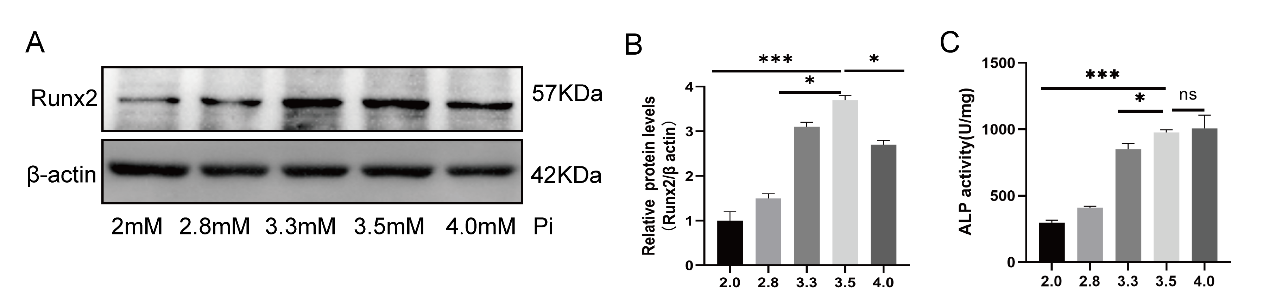


Supplemental Figure 3. Culturing VSMC in a high-phosphate medium will increase calcification in a dose-dependent manner. (A-C) VSMCs were incubated with 2.0 mM, 2.8 mM, 3.3 mM, 3.5 mM and 4.0mM respectively. (A, B) The expression of Runx2 was determined by Western blotting in VSMCs incubated with Pi. (C)ALP activity was measured by an ALP kit in VSMCs incubated Pi. Data are shown as mean ± SD, with three independent experiments. ns: no significance, **p* < 0.05, ***p* < 0.01, ****p* < 0.001.

Supplemental Figure 4


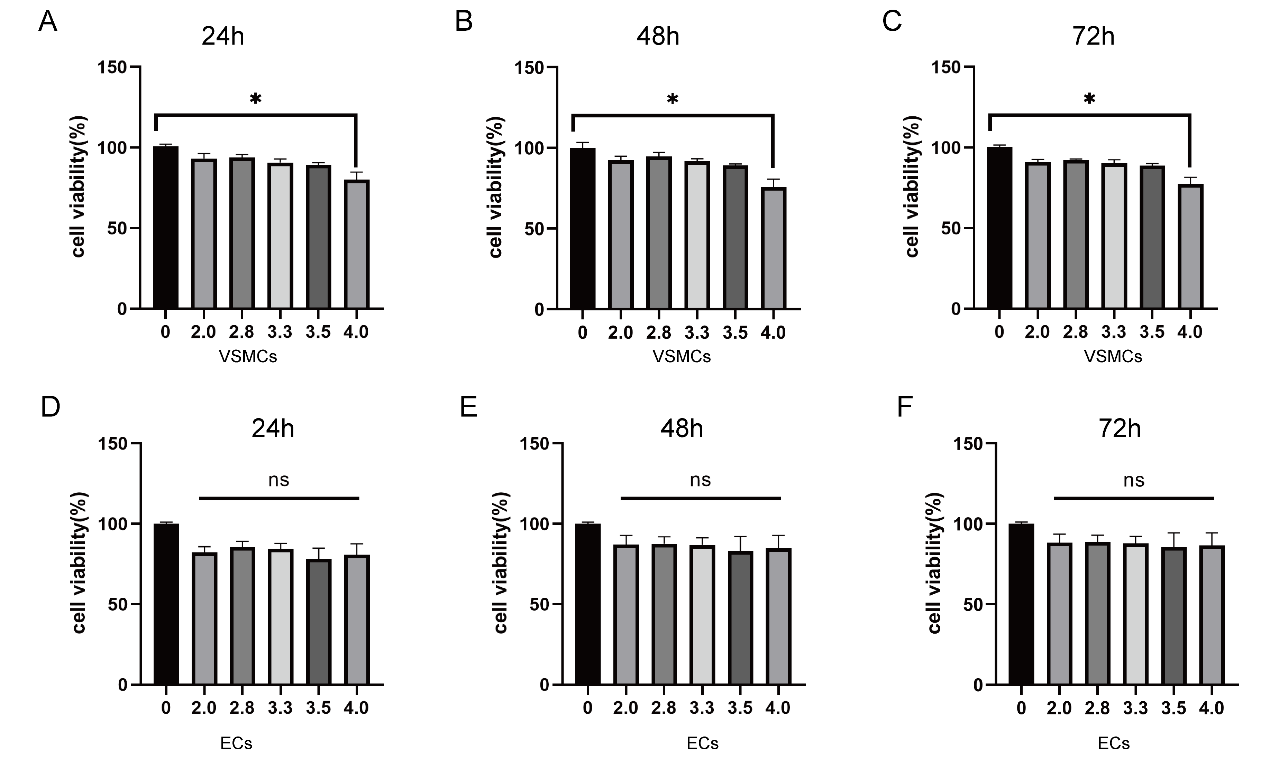


Supplemental Figure 4. Cell viability after incubation with different concentrations of phosphorus. (A-C) The cell viability of VSMCs treated with different concentrations of phosphorus cell viability was measured by CCK-8 kit on 24h,48h, and 72h. (D-F)

The cell viability of ECs treated with different concentrations of phosphorus; cell viability was measured by CCK-8 kit on 24h,48h, and 72h. Data are represented as the mean ± SD, with five replicates for each group. ns: no significance, **p* < 0.05, ***p* < 0.01.

Supplemental Figure 5


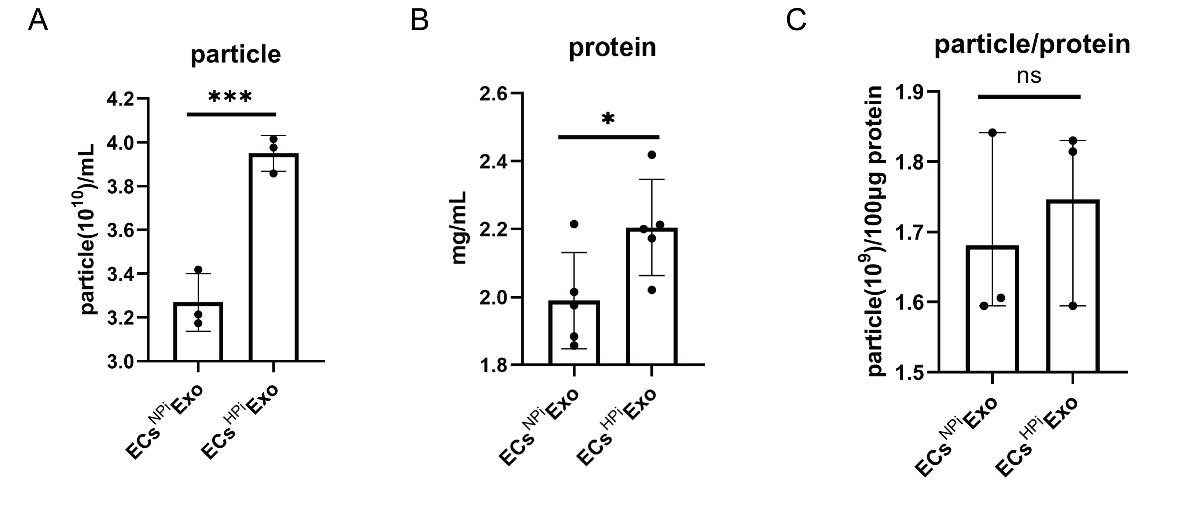


Supplemental Figure 5. Particle and protein yield of ECs^NPi^-Exos and ECs^HPi^-Exos.

(A) The protein yield per milliliter among ECs^HPi^ Exos and ECs^HPi^-Exos(n=5). (B) Number of particles isolated per milliliter among ECs^HPi^ Exos and ECs^HPi^-Exos(n=3). (C) Amount of particles per 100μg protein among ECs^HPi^ Exos and ECs^HPi^-Exos(n=3). Data are shown as mean ± SD. ns: no significance, **p* < 0.05, ****p* < 0.001.

Supplemental Figure 6


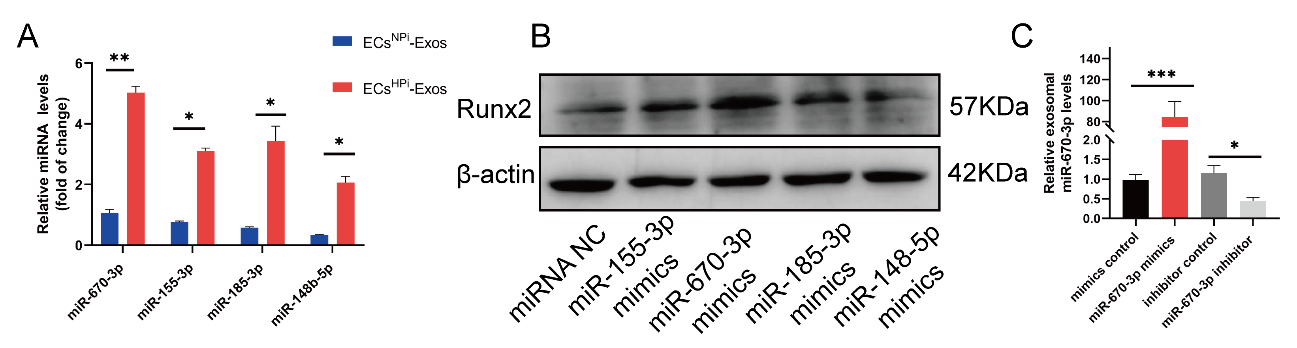


Supplemental Figure 6. (A) Expression level of miR-670-3p, miR-155-3p, miR-185-3p, and miR-148b-5p in ECs^NPi^-Exos and ECs^HPi^-Exos was analysed by qRT-PCR. (B) The expression level of Runx2 in VSMCs after being transfected with miR-185-3p, miR-155-3p and miR-148b-5p mimics was detected by Western blot. (C) qRT-PCR analysis of the level of miR-670-3p in exosomes transfected with miR-670-3p mimics and miR-670-3p inhibitor compared to those transfected with control oligos. Data are shown as mean ± SD, with three independent experiments. **p* < 0.05, **<0.01, ****p* < 0.001.

Supplemental Figure 7


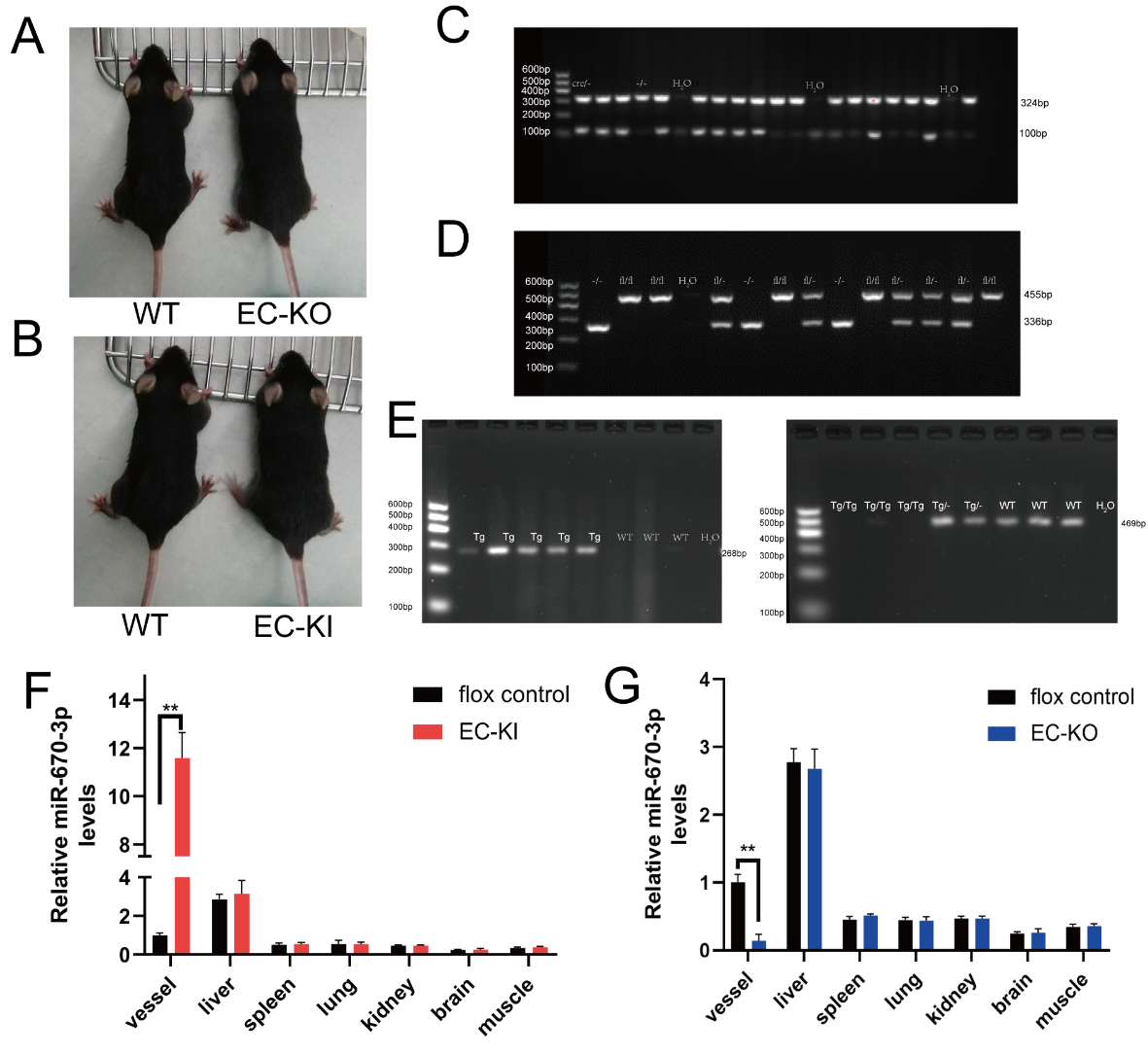


Supplemental Figure 7. (A,B) The generous appearance of the endothelial-specific miR-670-3p knock-in (miR-670-3p^EC-KI^) or endothelial-specific miR-670-3p knock-out (miR-670-3p ^EC-KO^) mice. (C) Agarose gel electrophoresis showed that Mice DNA with cre enzyme activity exhibited a 100bp mutate product beside the 324bp WT product. (D) Agarose gel electrophoresis showed that WT mice exert a single 336-bp PCR product, whereas both the 455-bp and 336-bp bands were observed with the DNA from miR-670-3p flox/+ heterozygous (fl/+) mice. and a 455-bp amplicon was obtained with the DNA from miR-670-3p flox homozygous (fl/fl) mice. (E) For ROSA26-CAG-LSL-miR-670-3p transgenic mice, the existence of mutant 268bp（left panel）with the exclusion of 469bp WT product（right panel）was identified as miR-670-3p knock-in homozygous(Tg/Tg) mice. (F) The expression levels of miR-670-3p in several organs of miR-670-3p ^EC-KI^ were detected by qRT-PCR. (G) The expression levels of miR-670-3p in several organs of miR-670-3p ^EC-KO^ mice were detected by qRT-PCR. Data are shown as mean ± SD, with three independent experiments. ***p*<0.01.

Supplemental Table 1

Primers for genotyping of mice

| Primer | Sequence (5’-3’) | Tm (℃) | Product  size (bp) |
| --- | --- | --- | --- |
| ROSA-GT-F | AGTCGCTCTGAGTTGTTATCAG | 57 | WT: 469  Mut:4467 |
| ROSA-GT-R | TGAGCATGTCTTTAATCTACCTCGATG | 58 |  |
| ROSA-GT-F | AGTCGCTCTGAGTTGTTATCAG | 57 | Mut:268 |
| ROSA-PCR-R | AGTCCCTATTGGCGTTACTATGG | 58 |  |
| 5'loxP-F | TGGAATTCAGCTGTGTGGCCTTCAT | 62 | WT:336  Mut:455 |
| 5'loxP-R | CCAGAATCTCCGGACTTGGCAGAAG | 62 |  |
| 3'TEK-F | GCGGTCTGGCAGTAAAAACTATC | 62 | WT: 324  Mut:100 |
| 3'TEK-R | GTGAAACAGCATTGCTGCTGCCACTT | 62 |  |

Note：WT: wide type allele； Mut: mutant allele

Supplemental Table 2

| Characteristic | Volunteers  (N=15) | CKD patients(N=15) | P-value |
| --- | --- | --- | --- |
| age | 37.42±11.56 | 40.25±10.96 | 0.479 |
| Male, n (%) | 8(0.53) | 8(0.53) | NS |
| Hypertension  , n(%) | 2(13.33) | 10(66.67) | 0.0078 |
| Cre(μmol/L) | 61.36±2.32 | 1182±152.2 | ＜0.001 |
| BUN（mmol/L） | 4.67±0.57 | 31.67±3.99 | ＜0.001 |
| UA(μmol/L) | 70.42±13.84 | 438.16±30.43 | ＜0.001 |
| csCa(mg/dl) | 8.26±1.28 | 9.45±1.35 | ＜0.001 |
| P (mg/dl) | 3.24±0.67 | 5.56±0.59 | ＜0.001 |
| Ca×P(mg^2^/dl^2^) | 26.52±2.73 | 52.49±3.27 | ＜0.001 |
| PTH(mmol/L) | 4.51±0.64 | 61.24±6.47 | ＜0.001 |
| 25(OH)D nmol/L | 50.32±5.12 | 35.21±4.37 | 0.006 |

Cre, creatinine; BUN, blood urea nitrogen; UA, uric acid; csCa, corrected serum

calcium; P, phosphorus; Ca × P, calcium-phosphorus product; PTH, parathyroid

hormone
